# Climate change adaptation and mitigation in African health systems: a scoping review of research and policy translation

**DOI:** 10.1136/bmjopen-2026-119188

**Published:** 2026-09-21

**Authors:** Mukhethwa Netshiombo, Óscar Brito Fernandes, Hans Ket, Nicolaas H Sperna Weiland, D Kringos

**Affiliations:** 1 Public and Occupational Health, Amsterdam UMC location University of Amsterdam, Amsterdam, Netherlands; 2 Quality of Care, Amsterdam Public Health Research Institute, Amsterdam, Netherlands; 3 Global Health, Amsterdam Public Health Research Institute, Amsterdam, Netherlands; 4 Amsterdam UMC Centre for Sustainable Healthcare, Amsterdam, Netherlands; 5 Medical Library, Vrije Universiteit Amsterdam, Amsterdam, Netherlands; 6 Department of Anaesthesiology, Amsterdam UMC, Amsterdam, Netherlands

**Keywords:** Legislation, Climate Change, Health Workforce, PUBLIC HEALTH

## Abstract

**Abstract:**

**Objectives:**

African health systems emit relatively low emissions yet experience disproportionate climate shocks. The objective is to map climate-health adaptation and mitigation research across African health systems and examine the translation of global climate and health commitments into domestic laws, policies and plans in the Southern African Development Community (SADC).

**Design:**

Scoping review, reported in accordance with the Preferred Reporting Items for Systematic reviews and Meta-Analyses extension for Scoping Reviews.

**Data sources:**

Six databases (OVID/Medline, Clarivate Analytics/Web of Science Core Collection, Embase.com, Elsevier/Scopus, Africa Index Medicus and Ebsco/CINAHL) were searched to obtain research literature. For the grey literature, one database (Climate Change Laws of the World) and targeted websites (SADC, national departments of health and environment, Alliance for Transformative Action on Climate and Health) were searched up to January 2025.

**Eligibility criteria:**

Studies addressing health systems in African countries and reporting on climate change adaptation and/or mitigation were included. Language and time restrictions were not applied. Studies that measured climate-related disease mortality and morbidity were excluded.

**Data extraction and synthesis:**

Data were extracted by one author and independently checked by another author. The data were thematically organised using a framework for building climate-resilient and low-carbon health systems.

**Results:**

We included 78 research and 15 grey literature publications. Research literature was predominant in two framework domains: climate-smart health workforce and climate-resilient and low-carbon infrastructures, technologies and supply chains. Only two SADC countries (South Africa and Mauritius) had a Climate Change Act, while four countries (South Africa, Tanzania, Madagascar and Zambia) had a national health adaptation plan. Madagascar was the only SADC country that had conducted a national public hospital greenhouse gas emission assessment.

**Conclusions:**

Immediate climate threats drive responses in African health systems to prioritise adaptation over mitigation in both research and governance. Stronger integration of climate objectives into health systems planning will be essential to support climate-resilient and sustainable low-carbon implementation.

**STRENGTHS AND LIMITATIONS OF THIS STUDY**

- Included literature was organised using the WHO operational framework for building climate-resilient and low-carbon health systems.
- Language restrictions were not applied to capture the diversity of regional language publications.
- Legislative and policy translation was conducted in SADC, limiting governance generalisability to the rest of the African continent.
- Policy documents may have been missed in the grey literature search due to limited public accessibility and inconsistent archiving or indexing.

## Introduction

 Climate change is becoming a critical driver of stress and disruption in the health system. Globally, health systems contribute an estimated 4–6% of total greenhouse gas (GHG) emissions,^1^ commonly classified into three scopes: Scope 1 covers direct emissions from sources owned or controlled by a health system (anaesthetic gases, transport fleet, boilers); Scope 2 comprises indirect emissions from purchased electricity; and Scope 3 includes other indirect emissions across the value chain, such as those associated with manufactured and purchased medical equipment and supplies.^2 3^

Health system emissions vary across regions, with high-income countries accounting for 6–8% of national carbon footprints,^4^ while low- and middle-income countries (LMICs) such as South Africa and Kenya account for less than 3% of national emissions.^5^ The current high carbon intensity and projected socio-economic expansion in these LMICs, however, will result in increased health system emissions over time.^6^

Despite their relatively low carbon-emitting contribution, African health systems are among the most vulnerable to climate-related shocks.^7^ Exacerbating factors include exposure to floods and heatwaves in systems with fragile capacity, infrastructure limitations, and constrained human and financial resources.^7 8^ The imbalance between low contribution and high vulnerability underscores the need for both climate adaptation (the process of adjusting to ‘actual or expected climate and its effects’) and mitigation (‘intervention to reduce emissions’) responses.^9^

Global climate change treaties and initiatives, including the Paris Agreement and the World Health Organization’s (WHO) COP26 health programme, have fostered political commitment toward climate-resilient and sustainable health systems.^10^ These binding and supportive programmes guide countries to develop and implement statutory and strategic priorities,^11 12^ however, translation into enforceable (sub)national climate laws, health sector policies, and operational plans can vary across countries. For example, policy analysis in South Africa documented incoherence in the translation of national climate and health adaptation directives into provincial policies and implementation plans.^13^ While there is a growing body of climate-health research and increasing engagement with global climate-health initiatives across Africa, the extent of translation into health sector legislation, policies, strategies and implementation plans remains unclear.

This study aims to (i) map emerging climate-health adaptation and mitigation research across Africa and (ii) assess the extent to which laws, policies and plans have been domesticated into health sector governance across the 16 member states of the Southern African Development Community (SADC): Angola, Botswana, Comoros, Democratic Republic of the Congo, Eswatini, Lesotho, Madagascar, Malawi, Mauritius, Mozambique, Namibia, Seychelles, South Africa, Tanzania, Zambia and Zimbabwe. By doing so, the study will identify key gaps, challenges and opportunities to inform future policy action and research. The following research questions were addressed:

1. What health adaptation and mitigation research trends are emerging to address climate change in Africa?
2. To what extent have global climate change agreements and commitments been translated into (sub)national climate laws and policies in SADC member states?
3. How have (sub)national climate change laws and policies been incorporated into health sector policies, strategies and plans in SADC member states?

## Methods

The review was conducted according to the Joanna Briggs Institute methodology for scoping reviews^14^ and reported in accordance with the Systematic Reviews and Meta-Analyses extension for Scoping Reviews guidelines.^15^ A two-pronged approach was applied to separately determine continental (Africa) emerging research trends and regional (SADC) translation of global climate commitment into related laws, policies, strategies and plans. Accordingly, a systematic research literature search was conducted for the former and a targeted grey literature search for the latter.

The SADC member states provide a relevant subregional case for examining gaps in translating global climate commitments into national health sector action. The member states face comparable climate-related risks but differ in health system capacity, governance arrangements and statutory maturity, enabling assessment of how similar global commitments are interpreted and operationalised across diverse national health system contexts.

### Eligibility criteria

Eligibility criteria were developed according to the Population–Concept–Context (PCC) framework for scoping reviews. The population/context comprised African health systems, defined as ‘organisations, people and actions whose primary intent is to promote, restore or maintain health’,^9^ while the concept was climate change adaptation and mitigation within health systems. Eligible sources included research literature (primary research, reviews, preprints, reports, editorials, commentaries, opinions and conference abstracts) and grey literature (laws, policies, strategies and plans). Sources were excluded if they focused solely on climate change-related health outcomes (eg, climate-related disease mortality and morbidity), if climate change laws, policies, strategies and plans did not include the health sector, or if documents had been superseded by a newer version. No restrictions were applied by publication date or language.

### Information sources and search strategy

A comprehensive search for the research literature was performed from inception in collaboration with a medical information specialist (JCFK). Initially, an explorative search was performed in OVID/Medline to identify relevant articles on the topic and refine search terms. This was followed by an expanded search across OVID/Medline (up to 2 December 2024), Clarivate Analytics/Web of Science Core Collection, Embase.com and Ebsco/CINAHL Plus with full text (up to 9 January 2025), and Elsevier/Scopus and Africa Index Medicus (up to 10 January 2025). The search employed controlled and free text terms for synonyms of ‘health care’ or ‘health facilities’ and ‘sustainable development’ or ‘low carbon’ or ‘climate change’ or ‘waste’ and ‘Africa’. Full search strategies are available in the online supplemental file 1. Duplicate articles were removed by JCFK using EndNote X20.0.1 (Clarivate), applying both the Amsterdam Efficient Deduplication and Bramer methods.^16 17^

Grey literature searches targeted climate change laws, policies, reports, strategies and plans produced by governments and key international organisations. Two complementary search strategies were employed: (1) a search of the Climate Change Laws of the World (CCLW) database using structured filters (country: SADC member states; document type: policy, law, UNFCCC submissions; sector: health), with screening of the first 200 results (approximately 10 pages) by title and summary; and (2) targeted website searches (online supplemental table 1) of the SADC, country-specific national departments of health and environment, and the Alliance for Transformative Action on Climate and Health (ATACH). The search result threshold (200 results and/or 10 pages) was pre-specified to balance comprehensiveness with feasibility, consistent with grey literature search protocols. For website searches, country-specific and scope-specific search terms (country names and/or ‘climate change’) were used within each website’s search function or document repository when available. When a website search function was unavailable, the organisational structure was navigated manually to locate climate and health documents.

### Selection process

To refine the eligibility criteria, 70 articles were randomly selected and independently screened by MN and ÓBF based on title and abstract. Discrepancies in inclusion, exclusion and undecided sources were discussed until consensus was reached. This informed the subsequent screening process.

**Figure 1:**
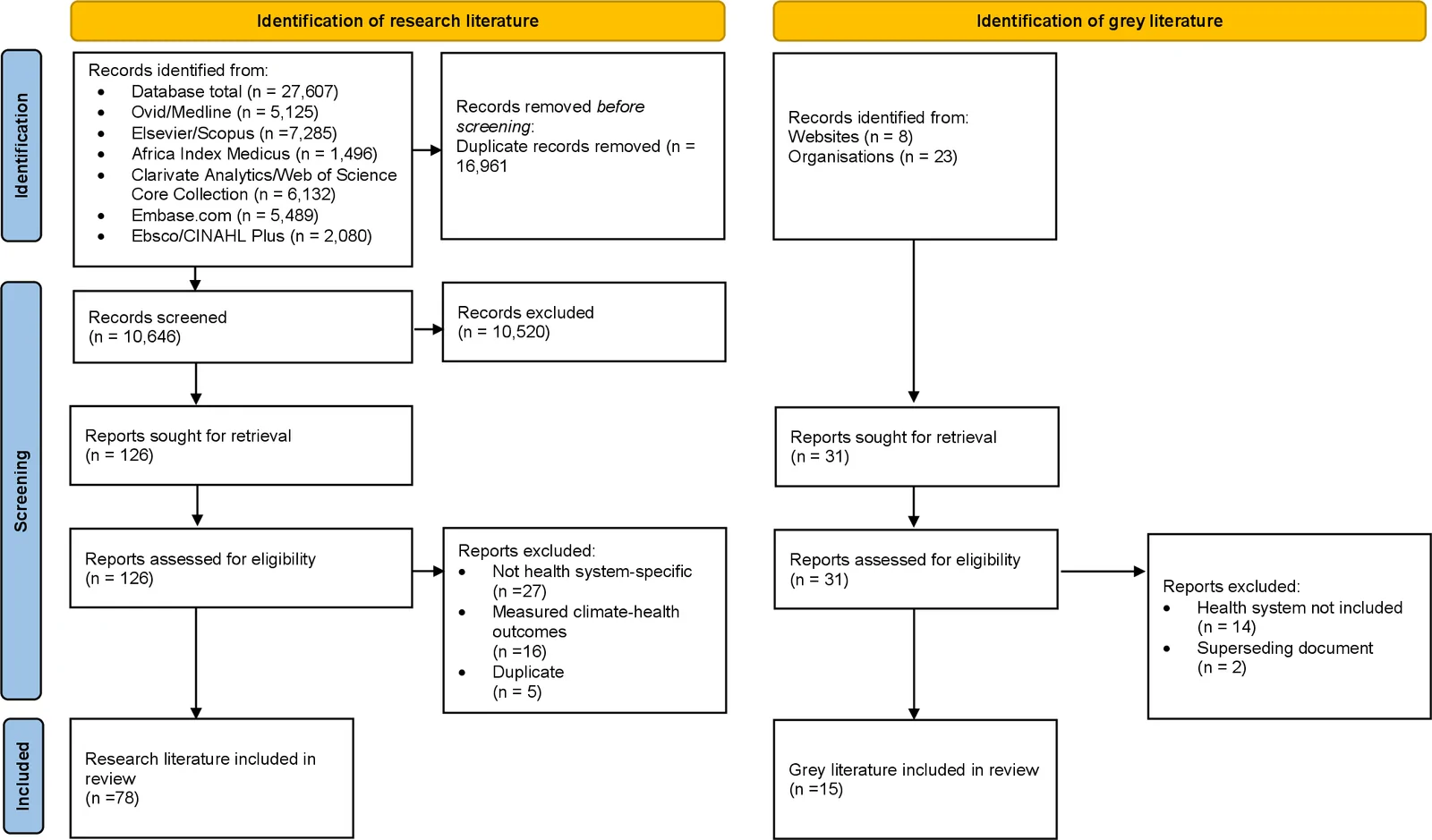
Adapted PRISMA diagram of research literature in Africa and grey literature in the SADC. PRISMA, Preferred Reporting Items for Systematic Reviews and Meta-Analyses; SADC, Southern African Development Community.

Research literature was managed and screened using Rayyan QCRI (Qatar, Doha).^18^ Title and abstract screening were conducted by MN, with a second reviewer (ÓBF) consulted to finalise ‘undecided’ sources for inclusion or exclusion using the predefined eligibility criteria. Full-text screening of peer-reviewed literature was conducted independently by two reviewers (MN and ÓBF), with discrepancies resolved through discussion. Grey literature was managed using Microsoft Excel. Where abstracts were unavailable, executive summaries were screened. Documents not published in English were translated using DeepL Translate (Germany, Cologne).^19^ The results of the search, including reasons for exclusion of sources and inclusion process, are reported in figure 1.

### Data charting process and synthesis

A structured data-charting form was developed to extract key information from the included sources. Data extraction was conducted by MN and independently checked by ÓBF. The literature was summarised and thematically organised using the WHO Operational Framework for Building Climate-Resilient and Low-Carbon Health Systems.^9^ The framework outlines 10 climate-focused domains aimed at providing direction for the health sector to respond effectively to climate-related health risks, advance low-carbon, sustainable healthcare delivery and delineate the roles and responsibilities of decision-makers in building climate-resilient health systems. For research literature, extracted data included study objectives, study design and key findings. For grey literature, a narrative content analysis was conducted to synthesise legislative and policy features.

Given the objective of mapping research evidence and policy translation across a broad and heterogeneous body of literature, no formal quality appraisal was undertaken, in line with Joanna Briggs Institute guidance for scoping reviews.^14^

### Patient and public involvement

None.

## Results

### Search results

The database search yielded 10 646 records (figure 1). Following title and abstract screening, 126 records were assessed in full text, of which 78 were included for review. 15 grey literature documents were identified and included for review from the CCLW database and targeted websites.

### Literature characteristics

The included research literature documents (online supplemental table 2) were published between 2006 and 2024, with most published from 2021 onwards. Research literature was predominantly multinational and/or continental (n=20),^20–37^ followed by country-specific studies in South Africa (n=17),^38–53^ Egypt (n=11)^54–64^ and Nigeria (n=8).^65–70^ Evidence from other African countries, including Ghana, Kenya, Uganda, Zimbabwe, Morocco, Zambia, Chad, Somalia, Mozambique, Ethiopia, Benin, Algeria, Rwanda and Senegal,^71–93^ was comparatively sparse, with fewer than four studies per country. Research literature publications included 56 original empirical studies, 8 reviews and 14 editorials, reports, commentaries, communications, position papers or perspectives. Research literature was predominantly mapped to two WHO framework domains—climate-smart health workforce and climate-resilient and low-carbon infrastructures, technologies and supply chain. Other literature was mapped to health and climate research, climate and health risks and GHG emissions assessments, climate-related emergency preparedness and management, integrated risks monitoring, early warning, and GHG emission tracking, and climate-informed health programmes. Key findings are presented in table 1. Grey literature was predominantly mapped to the transformative leadership and governance domain, with 11 of the 16 SADC countries represented (South Africa, Madagascar, Tanzania, Zambia, Mauritius, Botswana, Lesotho, Namibia, Zimbabwe, Angola, Malawi). Botswana, South Africa, Tanzania and Zambia had at least two climate and health-related laws, policies, strategies and/or plans (online supplemental table 3).

**Table 1:** Key findings of emerging research trends in Africa

WHO domains	Key findings
Climate-smart health workforce	Climate change is weakly integrated into Health Sciences curricula. Anaesthesiology, radiology and nursing provide illustrative cases for leadership-supported sustainable low-carbon practice integration into daily operations.
Climate-resilient and sustainable technologies, infrastructure and supply chain	Environmental, economic and energy security co-benefits incentivise investment in renewable and hybrid energy. Procurement officers' sustainability knowledge, not existing institutional protocol, drives green purchasing.
Climate and health risks and GHG emissions assessments	Extreme weather events disrupt access to care, however, traditional health practitioners contribute to continuity of care at community level.
Integrated risk monitoring, early warning and GHG emissions tracking	Meteorological and health data systems are mostly siloed with some integrated systems vulnerable to donor-funded support.

GHG, greenhouse gas.

### Emerging climate change adaptation and mitigation research in African health systems

#### Climate-smart health workforce

Studies demonstrated limited and uneven integration of climate change-related content into health professional education and training. Structured education for sustainable healthcare (ESH) and planetary health (PH) was scant across undergraduate and postgraduate curricula, with coverage ranging from 21% to 60% across African countries.^23 41 43 44 56 94^ In South Africa, 75% of anaesthesiologists reported no prior training on environmental sustainability.^41^ Where climate-related content was included, both formative and summative assessment methods were used, including written assignments, reflections, oral presentations and multiple-choice examinations.^44^

Educational interventions were associated with improvements in knowledge, attitudes and self-reported behaviours among medical students.^54^

Health professionals could identify climate-health gaps in their training,^35^ including limited preparedness to manage climate-related conditions such as acute malnutrition and re-emerging climate-sensitive diseases.^88 95^ Radiographers across 14 African countries reported exposure to sustainability training. However, integration into routine practice was limited, with only 39% reporting discussion of sustainability during work meetings.^94^ Across studies, healthcare workers acknowledged the relevance of the climate change-health nexus, although levels of knowledge varied substantially by setting and profession.^24 25 33 41 56 63 75 94 96^

Health professionals attributed facility-level mitigation efforts to organisational leadership and designated sustainability teams or climate activists.^48 59 94^ A green hospital leadership awareness programme encouraging staff to adopt energy-saving behaviours, such as switching off lights and appliances when not in use, was associated with reduction in the hospital carbon footprint over 5 years.^52^ Other mitigation efforts included adopting renewable energy, low-energy lighting, real-time power monitoring, energy-efficient heating, waste recycling and sustainable anaesthesia practices.^38 94^ Despite association guidelines to promote lower-impact anaesthesia techniques,^42^ environmental considerations were inconsistently applied in clinical decision-making.^41 47^ Reported barriers to mainstream climate action included limited leadership commitment, financial constraints, workforce awareness gaps and concerns regarding organisational climate priorities.^48 58^

#### Health and climate research

Climate and health research priorities included the need for transdisciplinary, solution-oriented and context-specific approaches. Research challenges included domestic funding constraints and reliance on external funding which limited locally-led evidence generatiom, weak institutional leadership, fragmented collaboration, and scarce data-capturing systems.^20 30 31 35 40^ Emerging research looked into opportunities from multi-sectoral collaboration between environmental, meteorological and health experts to support evidence-based responses to climate-sensitive diseases;^86 92 20^ evaluation of health system interventions and climate-impact assessments;^31^ the link between carbon emissions and climate-related healthcare costs;^66^ and climate change-induced migration challenges related to service delivery, equity, medicines and health financing access, and culturally appropriateness of health system responses.^20 27 53^

#### Climate-resilient and low-carbon infrastructures, technologies and supply chain

Simulation and techno-enviro-economic analyses from Algeria, Ethiopia, Kenya, Ghana, Egypt, Nigeria, Uganda, Morocco and Somalia demonstrated the potential of telemedicine, renewable and hybrid energy systems to improve patient experiences, reduce emissions, lower operating costs and enhance facility resilience, particularly in settings with unreliable electricity supply.^61 65 69–71 73 77 78 82 85 90 97^ Waste-to-energy and energy- and environmentally-efficient measures explored in Sub-Saharan and Egypt were largely at pilot or modelling stages.^29 39 55^ Implementation of green procurement in hospitals was influenced by buyers’ (procurement officers') sustainability knowledge^74^ and financial constraints,^46^ while medicine distribution drones were harnessed to reduce the environmental impact of medical supply chains.^77^

#### Climate-related emergency preparedness and management

Emergency preparedness assessments identified fragile governance and information-sharing systems, and limited integration of climate risks into facility-level disaster planning, including inadequate provisions for floods and continuity of chronic disease management.^40 49 84 87^ A multi-country initiative in Botswana, Mauritania, Niger, Nigeria and Togo demonstrated the feasibility of 24-hour rapid-response specialist teams for deployment during climate shocks.^22^

#### Climate-informed health programmes

In Egypt, nurse-led climate-health programmes were associated with improved patient management of chronic diseases.^64^ In South Africa, gaps in the availability and uptake of climate services tailored to health programme planning were linked to weak collaboration between health and meteorological institutions,^45^ while approaches to managing heat-vulnerable groups included governance-supported community engagement, outreach-based care and hospital infrastructure climate-proofing.^34^ In parallel, hospitals were implementing sustainable practices to reduce coal use and food waste.^52^

#### Climate and health risks and GHG emissions assessments

Identified risks included extreme weather events, service disruptions, damaged infrastructure, unreliable energy and water supplies, compromised cold-chain maintenance^80 83 91 98 99^ and constrained human and financial resources.^79 91^ Floods increased patient walking time and slowed referral transportation.^89^ In some instances, traditional health practitioners became the first point of consultation when extreme weather events disrupted access to healthcare facilities.^83 84^

High ambient temperatures and poor ventilation were associated with reduced health worker productivity, altered healthcare-seeking behaviour,^40 76^ higher energy demand for cooling medical equipment^72^ and climate-related disease burdens that strained fragile services in migrant-absorbing countries.^21^ In South Africa, a climate risk and vulnerability assessment framework was being developed to support national and local climate shock preparedness.^50^

Madagascar was the only country with a national public hospital carbon footprint assessment, covering Scopes 1–3 emissions, estimated at 68,603.660 tonnes of carbon dioxide equivalent (tCO₂e).^100^ Scope 3 accounted for the most emissions (57%), followed by Scope 1 (27%) and Scope 2 (16%). Facility-level assessments in Tanzania and Kenya identified supply chains, pharmaceuticals, medical and surgical supplies and laboratory consumables, chemicals and supplies as major contributors to Scope 3 emissions.^26^ In Morocco, a departmental carbon footprint assessment of haemodialysis attributed emissions to electricity use, procurement and staff and patient travel.^81^ Chronic obstructive pulmonary disease short-acting beta-agonist inhalers were identified as avoidable contributors to health sector emissions in Egypt (84,035.2 tCO₂e) and South Africa (102,181.6 tCO₂e).^32^

#### Integrated risks monitoring, early warning and emissions tracking

Integrated climate-health monitoring was constrained by limited routine surveillance capacity and weak reporting systems in Zimbabwe, Mozambique and Tanzania.^91 95 101^ District-level early warning systems for climate-sensitive diseases were reported in Benin and Ethiopia,^92 93^ while the prediction limitations of geospatial data were identified in Kenya and Botswana.^28^ Mitigation-related monitoring of a low-flow anaesthesia intervention showed a 30% carbon footprint reduction in a South African hospital.^38^

### Translation of global climate commitments into national climate laws and policies in SADC

**Figure 2:**
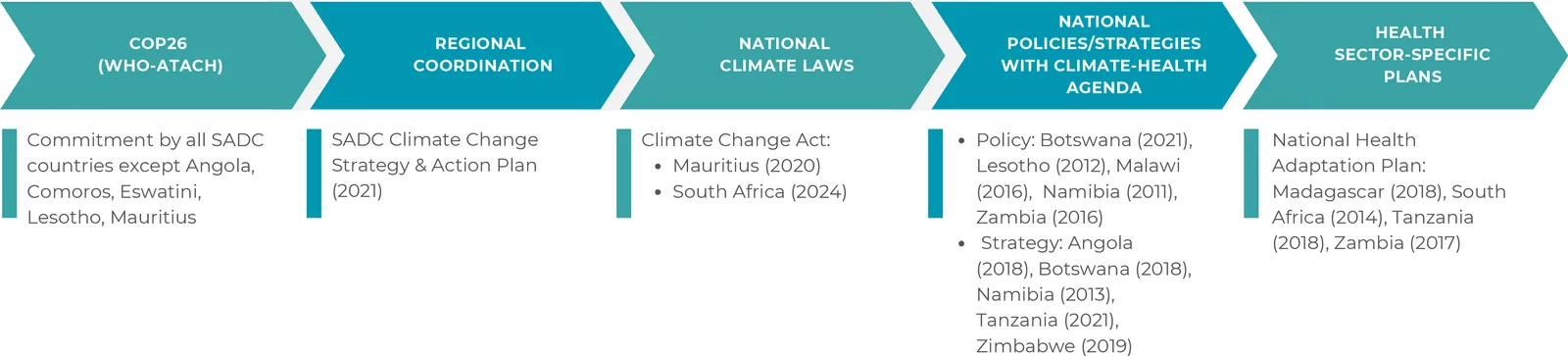
Translation of global climate commitments into national climate laws, policies, strategies and plans across SADC countries. ATACH, Alliance for Transformative Action on Climate and Health; SADC, Southern African Development Community.

SADC countries demonstrated varying degrees of global climate commitment domestication (figure 2). At a regional level, the SADC Climate Change Strategy and Action Plan provided overarching guidance for the health sector to strengthen research, facilitate climate responses and community resilience.^102^ Among the 16 SADC member states, only South Africa and Mauritius had enacted climate change legislation. Both climate change Acts established statutory obligations relevant to the health sector, including emission reduction targets and adaptation planning, supported by interministerial governance and reporting structures.^103 104^

Several SADC countries had adopted national climate change policies that include the health sector, however, governance and coordination structures were implicitly stated. Namibia’s policy addressed provision of water, sanitation and hygiene (WASH) in hospitals during floods to avoid climate-related disease, Botswana emphasised climate change research for evidence-based decision-making and community-informed climate action; Zambia’s policy inclusion of the health sector was limited to description of climate governance structutures (council of ministries and an advisory steering committee); and Lesotho’s policy was the only one to included GHG emission reduction as a national objective but policy actions to achieve this were not specified.^105–107^

### Incorporation of climate change into health sector strategies and plans in SADC

Integration of health into national and local climate change adaptation plans in South Africa was described as inadequate^51^ and centralised governance bureaucracy was considered an impediment to local climate action in Nigeria.^61^ Six SADC countries had adopted climate change strategies or action plans that include health-related objectives, either as stand-alone guidance documents or as instruments translating policy aims into operational measures. Specificity varied, with Zimbabwe defining time-bound targets and estimating adaptation costs,^108^ while Botswana’s policy highlighted the need for legislative ammendments to include climate change laws. Other countries articulated short- to long-term objectives without clear timelines, health sector resilience responses that include infrastructure rehabilitation, capacity-building measures such as climate and health training and research development, and strengthening adaptive capacity, including disease surveillance, emergency preparedness and early warning systems.^109–113^

Within the health sector, national health vulnerability and adaptation assessments were conducted in a limited number of countries, including Tanzania, Madagascar, Malawi, Zambia and Mozambique.^91 98 99 101 114 115^ The assessments profiled climate-sensitive health risks and outlined monitoring and reporting frameworks, with some including cost estimates and proposed financing arrangements, while implementation feasibility and funding mechanisms remained uneven.^91 98 99 114^ Across all SADC member states reviewed, low-carbon or sustainable healthcare system action plans were not identified, however Madagascar had performed a GHG emission assessment. Suggested mitigation measures included energy transition, improved medical and non-medical waste management, promotion of virtual consultations and reduced flight use for staff travel. Finances were identified as both a potential savings co-benefit of and constraint to mitigation implementation.^100^

Four SADC countries (Madagascar, South Africa, Tanzania and Zambia) had developed Health National Adaptation Plans,^101 115–117^ with Madagascar and South Africa demonstrating relatively strong governance arrangements, while other plans lacked clarity regarding roles, accountability, and coordination mechanisms.

## Discussion

This scoping review explored health system-level climate change adaptation and mitigation research trends in Africa and examined how global climate commitments have translated into national laws and health sector-specific policies and plans in SADC. Across research and policy, a consistent pattern emerged: adaptation dominates climate responses, while mitigation and health system decarbonisation remain limited and variably operationalised.

At the continental level, mainstreaming of climate change into health professionals’ education and training was scant and uneven.^33 35 44^ While health workers generally acknowledged the climate-health nexus,^41 56^ the capacity to manage related health risks and implement sustainable practices was insufficient,^25 88 95^ reflecting fragmented curricular integration rather than a lack of climate awareness. Institutionalisation of climate education accross health training programmes in the USA ^118^ is contrasted by gradual but promising integration in South Africa,^44^ where knowledge platforms and exchange of good practices among ESH and PH educators is proposed as a measure to mainstream climate into curricula and training.^23 119^

Professional ‘green teams’ and individual champions were driving facility-level change, particularly in radiology, nursing and anaesthesiology, but their impact depended on leadership support and organisational mandate,^46 52 59^ underscoring the potential and limits of individual-led initiatives.^120^

Health system GHG emissions were documented across all three emission scopes: facility-level studies identified Scope 1 emissions from anaesthetic gases,^38^ haemodialysis^81^ and generators;^65 70 90^ scope 2 emissions from electricity-intensive equipment; and scope 3 emissions were dominated by procurement and pharmaceuticals.^26 32 100^ Madagascar was the only country with a reported national assessment of the public health sector’s emissions which account for less than 1% of total national contributions (29,584.6 kilotonnes of carbon dioxide equivalent (ktCO₂e)).^100^ The carbon footprint is lower than in counterpart SADC countries, including South Africa, Kenya and Mauritius.^5^ A contributing factor may be the exclusion of private health facilities from the assessment and the comparatively lower scale and complexity of healthcare service delivered compared to other countries.^100^

Identified mitigation efforts were discipline- and region-specific, including lower carbon impact anaesthesia practices in South Africa^38 42 47^ and techno-enviro-economic modelling for transition to hybrid and renewable energy systems in Nigeria, East (Ethiopia, Somalia and Uganda) and North (Egypt, Morocco and Alegeria) Africa.^61 65 69–71 73 82 85 90 97^ While these interventions offer a dual benefit of reducing emissions and strengthening service continuity during climate shocks, confinement to modelling can lead to a ‘pilot trap’ in which mitigation strategies do not progresses to scale.^121^ Fiscal and institutional constraints, such as those documented in Egypt’s green transition efforts, appear to be a key limiting factor in this regard.^122^

Across sub-Saharan Africa, responses to extreme weather events prioritised emergency preparedness, disease surveillance and service delivery resilience.^92 93^ This adaptation-led emphasis reflects rational prioritisation in fragile systems facing acute climate shocks. That said, opportunities to anticipate and respond to climate-sensitive health risks such as vector-borne disease outbreaks and extreme weather events remained under-used, with traditional health practitioners emerging as a well-placed workforce to respond to community-level climate action.^22 28 51 83 84 88 95^ The absence of structured collaboration between meteorological services and health institutions further constrained the development of evidence-informed climate-resilient health programmes.^45 51^

SADC member states’ translation of international climate commitments into domestic laws and policies varied. South Africa and Mauritius had enacted climate change legislation with explicit relevance to the health sector.^103 104^ Integration of climate change into health system plans across SADC countries was fragmented, owing in part to limited leadership accountability, unclear role delineation and poor coordination across governance levels.^123^ Health National Adaptation Plans were emerging, with limited attention toward low-carbon and sustainable health system action planning.^124^ Although legislation enables action, experiences from Italy and Brazil illustrate how ineffective governance can stall implementation,^125^ while the United Kingdom and Netherlands demonstrate how strong institutional capacity can facilitate policy execution.^126 127^ In some countries, limited legal and institutional capacity may drive the development of non-binding policies and strategies as more flexible and less resource-intensive instruments for climate action. Unfortunaately, these lack enforceability and prioritisation can be vulnerable to political cycles.^128 129^

Overall, adaptation dominated research and policy responses. Acute climate shocks, fragile service capacity and competing health priorities incentivise reactive adaptation, while mitigation is deprioritised due to limited resources and the relatively low carbon footprint of African health systems.^130^ That said, adaptation strategies that do not integrate sustainability risk reinforcing carbon-intensive practices and creating negative feedback loops that may increase long-term emissions and undermine resilience.^10^ Considering the high carbon intensity of health systems in LMICs, aligning short-term resilience with long-term sustainability is therefore essential.

### Strengths and limitations

The strengths of this study include the use of the WHO Operational Framework for Building Climate-Resilient and Low-Carbon Health Systems to thematically synthesise both continental research trends and regional (SADC) policy developments. To accommodate the regional language diversity and avoid bias, there were no language restrictions applied.

This study has limitations. Title and abstract screening were primarily undertaken by one reviewer. To reducce the risk of missed records, inclusion criteria calibration of 70 articles was conducted with a second reviewer. While research evidence was mapped across Africa, the analysis of legal and policy translation was limited to SADC member states. Consequently, the policy findings may not be representative of other African regions. Although we undertook targeted grey literature searches, not all relevant policy documents may be publicly accessible, consistently archived or indexed, and therefore some materials may have been missed. In line with scoping review methodology, no formal quality appraisal was conducted, limiting the assessment of evidence robustness.

## Conclusions

Climate action within African health systems is advancing but remains fragmented and predominantly adaptation-led. While mitigation integration and statutory mechanisms are underdeveloped, emerging infrastructure and energy initiatives demonstrate feasible pathways toward low-carbon healthcare. Bridging adaptation and mitigation within health sector governance will be critical to achieving health systems that are both climate-resilient and environmentally sustainable.

Future research should consider how leadership can be leveraged to support facility-level climate action and develop systematic approaches to integrating health and climate across governance levels.

## Supplementary material

10.1136/bmjopen-2026-119188 online supplemental file 1

10.1136/bmjopen-2026-119188 online supplemental file 2

10.1136/bmjopen-2026-119188 online supplemental file 3

10.1136/bmjopen-2026-119188 online supplemental file 4

## Data Availability

All data relevant to the study are included in the article or uploaded as supplementary information.
