# Supplementary material for: Climate change adaptation and mitigation in African health systems: a scoping review of research and policy translation
Source: BMJ Open. 2026 Sep 21;16(9):e119188. doi: 10.1136/bmjopen-2026-119188 (PMC13599907; doi:10.1136/bmjopen-2026-119188)
Supplement: Supplementary file 1 [file bmjopen-2026-119188.reviewer_comments.pdf]

PEER REVIEW HISTORY

BMJ Open publishes all reviews undertaken for accepted manuscripts. Reviewers are asked to complete a checklist review form and are provided with free text boxes to elaborate on their assessment. These free text comments are reproduced below.

ARTICLE DETAILS

Title (Provisional)

Climate change adaptation and mitigation in African health systems: a scoping review of research and policy translation

Authors

netshiombo, mukhethwa; Brito Fernandes, Óscar; Ket, Hans; Sperna Weiland, Nicolaas H; Kringos, D

VERSION 1 - REVIEW

**Reviewer**	**1**
**Name**	**Chironda, Geldine**
**Affiliation**	**Seed Global Health**
**Date**	**03-May-2026**
**COI**	**none**

CLIMATE CHANGE ADAPTATION AND MITIGATION IN AFRICAN HEALTH SYSTEMS: A SCOPING REVIEW OF RESEARCH AND POLICY TRANSLATION

- **The context was on SADC; hence the topic should read as: Climate change adaptation and mitigation in** Southern African Development Community (SADC) African health systems**: a scoping review of research and policy translation**

ABSTRACT

- Objectives: Health systems in Africa are disproportionately vulnerable to climate risks, despite their greenhouse gas emissions being lower than the global average, creating a dual imperative for adaptation and mitigation. The sentence is not an objective, it is related to background information, hence need to create another subheading of the abstract which talks to this.

- Design: Rather call this methods or methodology.

- A two-pronged search strategy was applied: at continental-level (Africa) systematic literature search (OVID/Medline, Clarivate Analytics/Web of Science Core Collection, Embase.com and Ebsco/CINAHL) and at the regional level (SADC) targeted grey literature search (Climate Change Laws of the World database, the SADC website, national departments of health and environment, and the ATACH), up to January 2025. This is a very long sentence and the intended meaning is diminished. Kindly make this into 2 sentences.
- There is need to include what guided the search from data bases, that is the P (Africans), C (Climate change adaptation; mitigation), C (Health systems)
- Results: There is need to include the nature of the evidence found in terms of the type of methodologies, yearly trends and the origin
- Keywords: Africa, Carbon footprint, Decarbonising healthcare, GHG emissions, Resilience, Preparedness, Health Policy. Key words should originate from the topic. Kindly remove Carbon footprint, Decarbonising healthcare, GHG emissions, Resilience, Preparedness, Health Policy as key words. Kindly add Climate change adaptation, mitigation, health systems, research and policy translation as key words for this review.

INTRODUCTION

- Despite their relatively low carbon-emitting contribution, African health systems are among the most vulnerable to climate-related shocks. Kindly reference this claim
- Global climate change commitments and initiatives, including the Paris Agreement and the COP26 World Health Organization's (WHO) health program to integrate health into climate action, have fostered political commitment to climate-resilient and sustainable health systems. Kindly reference this claim
- In Africa, a growing body of research addresses climate-sensitive health risks, emission hotspots, and facility-level resilience, yet it remains unclear how this evidence is reflected in health sector governance and policy responses. Additionally, while some African countries are engaging with global climate-health initiatives, the extent to

which these engagements result in concrete health sector legislation, strategies, or implementation plans is not adequately captured. Kindly reference these two claims.

- This study aims to assess how African health systems are responding to climate change by mapping emerging climate change adaptation and mitigation research at the continental level and evaluating the development and integration of climate-related laws, policies, and plans within health sector governance across the 16 member states of the Southern African Development Community (SADC). The review aim is not exactly tallying with the topic even in terms of the context of the review. Secondly the study aim seems to be very long. Kindly rephrase the aim of reviews as follows: The review aimed at mapping the evidence on climate change adaptation and mitigation in 16 member states of the Southern African Development Community (SADC) African health systems in order to promote research and policy translation.
- There is need to include a question on the publication characteristics as the first question on revie questions

METHODOLOGY

- A scoping review protocol needs to be developed and registered in either open science framework or fig share. Therefore, was this protocol registered in any of these sites and kindly provide a link to the registration.
- A two-pronged search strategy was applied, including a systematic literature search at the continental (Africa) level to determine research trends, and a targeted grey literature search at the regional level (SADC) to map climate related laws, policies, strategies, and plans relevant to health systems. This is a very long sentence and the intended meaning is not clear. Secondly, this sentence does not belong here, it should be put under search strategy.
- The SADC - comprising 16 member states (Angola, Botswana, Comoros, Democratic Republic of the Congo, Eswatini, Lesotho, Madagascar, Malawi, Mauritius, Mozambique, Namibia, Seychelles, South Africa, Tanzania, Zambia, and Zimbabwe)- The details of the specific countries should be moved to where the context of the review is being described.
- Eligibility criteria: This section needs a complete revamp so that it is clearly stated for the readers to understand. Eligibility criteria is defined by inclusion and exclusion

criteria. Thus, the eligibility criteria should be expressed in terms of Population, Concept and Context- PCC. This is better expressed in terms of the following table:

Table 1:

	**Inclusion criteria**	**Exclusion criteria**
Population		
Concept		
Context		
Types of articles		

- Information source and search strategy: Although the search strategy was described, the process of selection is unclear. Meaning, a three-search strategy as defined by JBI scoping review methodology is not well elaborated. Kindly describe the 3-step search strategy so that this section becomes clear.
- Information source and search strategy: The search included controlled and free text terms for synonyms of ‘health care' or 'health facilities' and 'sustainable development' or 'low carbon' or 'climate change' or 'waste' and 'Africa'. The search words are not comprehensive. Words like climate change adaptation, mitigation, health systems, SADC and specific 16 countries within SADC should be the initial search words to be considered.
- Information source and search strategy: The search was performed without restrictions for methodology, date or language. This has been said in one of the subheadings and why is it being repeated?
- Information source and search strategy: What was the role of the librarian in the search strategy?
- Selection Process: Information on whether the duplicate were removed or not is not indicated in this section. Kindly indicate.
- Selection process; What was the role of the librarian in this process
- Selection process: How was the reporting of the whole selection process done?
- Data charting process: Rather call this subheading Data charting process and synthesis because this is what is indicated in the content

- Data charting process: For peer-reviewed literature, extracted data included study aims, objectives and key findings. Between the title and objective, may you please choose 1. Key findings should be tallied to answer the review questions.

RESULTS

- The combined scientific and grey literature search yielded 15,365 records (Figure 1). Following title and abstract screening of peer-reviewed literature and executive summary screening of grey literature, 168 documents were assessed in full text. 85 peer-reviewed publications and 15 grey literature documents were included, while 65 documents were excluded at the full-text stage. Kindly add a subheading for this content called **Search results**. While the authors tried to describe the results of the search process, there is need to include the reasons for exclusion at each stage of selection.

- The included documents were published between 2006 and 2024, with most published from 2021 onwards. Studies were predominantly multinational (n=22), followed by country specific studies focused on South Africa (n=16), Egypt (n=12), and Nigeria (n=8). Evidence from other African countries, including Ghana, Kenya, Uganda, Zimbabwe, Morocco, Zambia, Zimbabwe, Chad, Somalia, Mozambique, Ethiopia, Benin, Algeria, Rwanda, and Senegal, was comparatively sparse, with four or fewer studies per country. Among peer reviewed publications, 62 reported original empirical studies, 22 were reviews, and two were editorials or perspectives. Kindly indicate the subheading for this content as publication characteristics

- The first part of results section was quantifying the results (for instance n=5) while the rest of the sections brought in references. There is need to maintain uniformity.

DISCUSSION

- At the continental level, we found limited and uneven mainstreaming of climate change into health professionals' education and training. Kindly reference the documents from the identified studies talking to this.

- Accordingly, Zolo (2024) and Al-Adawi (2022) propose the creation of knowledge platforms and the exchange of good practices among educators in institutions of higher learning to integrate ESH and PH into curricula and training. Intext referencing for the rest of the document is in terms of numbers. Kindly correct this.

- Professional ‘green teams’ and individual champions have driven facility-level change, particularly in radiology, nursing and anaesthesiology, but their impact depends on leadership support and organisational mandate, underscoring the limits of individual-led initiatives without system-level commitment. There is need to support this claim with a reference

- African healthcare systems are among the most climate-vulnerable globally, as reflected in the predominant focus on adaptation. Across sub-Saharan Africa and SADC countries, responses prioritised emergency preparedness, disease surveillance and service delivery resilience to extreme weather events. This adaptation-led emphasis reflects rational prioritisation in fragile systems facing acute climate shocks, rather than policy neglect. That said, opportunities to anticipate and respond to climate-sensitive health risks such as heatwaves, vector-borne disease outbreaks, and extreme weather events remained under utilised. All these claims need to be referenced.

- An assessment of SADC countries found uneven translation of international climate commitments into domestic law and policy. While the SADC climate change strategy and action plan provide a regional framework, only South Africa and Mauritius have enacted binding climate change legislation with explicit relevance to the health sector. Kindly reference these claims

- Health National Adaptation Plans focus on vulnerability assessment and preparedness, while there is limited attention toward low-carbon and sustainable health system action planning. Kindly reference these claims

- Overall, adaptation dominates research and policy responses, while healthcare system mitigation remains marginal. Acute climate shocks, fragile service capacity and competing health priorities incentivise reactive adaptation, while mitigation is deprioritised due to limited resources and the relatively low carbon footprint of African health systems. Kindly reference this claim

---

Reviewer **2**

Name **LaRoche, Jason**

Affiliation **Janssen Research and Development LLC, Janssen Clinical Innovation**

Date **18-Jun-2026**

COI **none**

Dear Dr. Mukhethwa Netshiombo,

Thank you for submitting this manuscript for review. Your topic is extremely relevant and addresses a major gap in our current knowledge and understanding of the climate impact of healthcare, particularly in Africa.

While your paper is strong, it does require some revisions to ensure reproducibility and the robustness of your conclusions. After a careful review of your paper, I have the following suggestions organized sequentially by section:

Methods

Page-6: your grey literature limited to SADC while your research literature includes all Africa. This creates an asymmetry between policy and research results. This should be explained in your design choices.

Information Sources and Search Strategy

Page-7, line-58: "Documents were identified through hand-searching by country name, with titles and accompanying summaries screened within the first 10 pages of results." First 10 pages of results alone is not reproducible. Add the specific search engines/databases, search strings, and inclusion thresholds used.

Selection Process

Page-8: "Title and abstract screening were conducted by one reviewer (MN), with a second reviewer (ÓBF)consulted in cases of uncertainty." This deviates from standard practice as usually dual independent screening is expected to avoid bias. This should be acknowledged in the paper or the language softened to account for this.

Results

Page-9: In this section you note 15,365 records → 168 full-text → 85 + 15 included = 100; but when I look at Figure-1, the numbers do not align. The sum of records screened for both peer-reviewed and grey literature is 15,275. Total records included in the peer review is 90 in Figure-1. Please correct this discrepancy.

Page-11, lines 3-7: you repeat public health expenditure modeling twice consecutively.

References

I checked your citations and in your references you have two recorded under #25:

25. Irlam J, Razzack Z, Rother HA,...

25. Mohamed MAES, Ghallab E,...

I believe the latter should be #26 in your list of references. Scanning the text, I did not see any citations for #26. You'll likely need to double-check your citations under #25 and delineate those intended for #26 and update accordingly.

You have duplicate references under #5 and #98 as the same paper appears under both.

For references #65 (HNAP) and Ref #72 (carbon footprint report), both point to the same ATACH URL (HNAP 2018). Please update #72 with a link to the correct document.

You are missing a DOI for #35. The listed DOIs did not work for citations #43 and #54. For citation #84, the url does not work. A minor comment is that you are missing the "d" and just have "oi" in citation #39.

You include some pre-prints under references #31 and #41. As these have not yet been peer-reviewed, this should be acknowledged.

Thank you again for the strong efforts of you and your team. I believe that with revisions, this paper will be very valuable and contribute strongly to the current scientific knowledge.

Kind Regards,

Jason LaRoche

Director, Innovative Health - Environmental Sustainability, Johnson & Johnson Innovative Medicine

Faculty Member - Delft University of Technology

VERSION 1 - AUTHOR RESPONSE

Comments from Reviewers

Reviewer 1

Comments to the Authors: Kindly receive the comments attached.

CLIMATE CHANGE ADAPTATION AND MITIGATION IN AFRICAN HEALTH SYSTEMS: A SCOPING REVIEW OF RESEARCH AND POLICY TRANSLATION

The context was on SADC; hence the topic should read as: Climate change adaptation and mitigation in Southern African Development Community (SADC) African health systems: a scoping review of research and policy translation

Response: The continental (African) framing in the title is intentional, as the study has a two-pronged design: it maps climate-health research across Africa while examining the translation of global climate commitments into laws, policies and plans specifically within SADC member states. To make this distinction clearer, we have revised the Methods section to explicitly describe this two-pronged approach.

ABSTRACT

Objectives: Health systems in Africa are disproportionately vulnerable to climate risks, despite their greenhouse gas emissions being lower than the global average, creating a dual imperative for adaptation and mitigation. The sentence is not an objective, it is related to background information, hence need to create another subheading of the abstract which talks to this.

Response: We agree that the opening sentence provides background rather than stating the study objective. However, BMJ Open uses a structured abstract with predefined headings and does not include a separate background section. We therefore retained this brief contextual sentence under the Objectives heading to introduce the rationale for the study before stating the study objectives.

Design: Rather call this methods or methodology.

Response: We retained the subheading Design, as this follows BMJ Open's required structured abstract format, which specifies the headings Objectives, Design, Data sources, Eligibility criteria, Data extraction and synthesis, Results and Conclusions.

A two-pronged search strategy was applied: at continental-level (Africa) systematic literature search (OVID/Medline, Clarivate Analytics/Web of Science Core Collection, Embase.com and Ebsco/CINAHL) and at the regional level (SADC) targeted grey literature search (Climate Change Laws of the World database, the SADC website, national departments of health and environment, and the ATACH), up to January 2025. This is a very long sentence and the intended meaning is diminished. Kindly make this into 2 sentences.

Response: We agree that the sentence is lengthy and have therefore restructured it to provide clarity on the search strategy approach. The revised text now reads: Six databases (OVID/Medline, Clarivate Analytics/Web of Science Core Collection, Embase.com, Elsevier/Scopus, Africa Index Medicus and Ebsco/CINAHL)) were searched to obtain research literature. For the grey literature, one database (Climate Change Laws of the World database) and targeted websites (SADC, national departments of health and environment, ATACH) were searched up to January 2025.

There is need to include what guided the search from data bases, that is the P (Africans), C (Climate change adaptation; mitigation), C (Health systems)

Response: We agree that the eligibility criteria underpinning the search strategy were not sufficiently explicit. We have therefore revised this section to clarify the Population, Concept and Context (PCC) elements that informed the search strategy and eligibility criteria. We also clarified that the population element refers to studies conducted within the African continent, rather than African populations per se.

Results: There is need to include the nature of the evidence found in terms of the type of methodologies, yearly trends and the origin

Response: We agree that a clearer description of the included evidence strengthens the Results section. We have therefore expanded the Literature characteristics subsection in the Results to include publication trends over time, the geographical distribution of the included studies, and the methodological characteristics of the evidence. Additional details are provided in Supplementary Materials (Tables 2 and 3).

Keywords: Africa, Carbon footprint, Decarbonising healthcare, GHG emissions, Resilience, Preparedness, Health Policy. Key words should originate from the topic. Kindly remove Carbon footprint, Decarbonising healthcare, GHG emissions, Resilience, Preparedness, Health Policy as key words. Kindly add Climate change adaptation, mitigation, health systems, research and policy translation as key words for this review.

Response: Thank you for this suggestion. We have revised the keywords to better reflect the main concepts addressed in the review. The revised keywords are Africa; Climate-resilient health systems; Climate change adaptation; Climate change mitigation; Sustainable healthcare; Scoping review.

INTRODUCTION

Despite their relatively low carbon-emitting contribution, African health systems are among the most vulnerable to climate-related shocks. Kindly reference this claim

Response: The statement has been referenced: [7] - DOI:10.1016/S0140-6736(22)01540-9.

Global climate change commitments and initiatives, including the Paris Agreement and the COP26 World Health Organization's (WHO) health program to integrate health into

climate action, have fostered political commitment to climate-resilient and sustainable health systems. Kindly reference this claim

Response: The statement has been referenced: [10] DOI:10.2471/BLT.23.290464.

In Africa, a growing body of research addresses climate-sensitive health risks, emission hotspots, and facility-level resilience, yet it remains unclear how this evidence is reflected in health sector governance and policy responses. Additionally, while some African countries are engaging with global climate-health initiatives, the extent to which these engagements result in concrete health sector legislation, strategies, or implementation plans is not adequately captured. Kindly reference these two claims.

Response: We agree that this paragraph could be interpreted as making factual claims. Our intention was to articulate the knowledge gap that motivated the review rather than present evidence-based findings. We have therefore revised the paragraph to more clearly frame it as a gap in the existing literature and supported the preceding contextual statements with appropriate references [13]. The revised text now reads: While there is a growing body of climate-health research and increasing engagement with global climate-health initiatives across Africa, the extent to which this evidence has been translated into health sector legislation, policies, strategies, and implementation plans remains unclear.

This study aims to assess how African health systems are responding to climate change by mapping emerging climate change adaptation and mitigation research at the continental level and evaluating the development and integration of climate-related laws, policies, and plans within health sector governance across the 16 member states of the Southern African Development Community (SADC). The review aim is not exactly tallying with the topic even in terms of the context of the review. Secondly the study aim seems to be very long. Kindly rephrase the aim of reviews as follows: The review aimed at mapping the evidence on climate change adaptation and mitigation in 16 member states of the Southern African Development Community (SADC) African health systems in order to promote research and policy translation.

Response: The continental (African) framing of the title and study aim is intentional, as the review has a two-pronged design: it maps climate change adaptation and mitigation research across Africa while examining the translation of global climate commitments into health sector laws, policies and plans specifically within SADC member states. We agree that the original study aim was lengthy. We have therefore restructured it to clearly distinguish the

two components of the review: (i) mapping climate-health research across Africa and (ii) examining policy translation within SADC member states, thereby improving readability.

There is need to include a question on the publication characteristics as the first question on revie questions

Response: We agree that publication characteristics are an important component of a scoping review. These details have therefore been included in the Results section and Supplementary Tables 2 and 3. However, we have not framed them as a separate research question, as this was not one of the primary objectives of the review.

METHODOLOGY

A scoping review protocol needs to be developed and registered in either open science framework or fig share. Therefore, was this protocol registered in any of these sites and kindly provide a link to the registration.

Response: We did not prospectively register a protocol for this scoping review. While protocol registration is encouraged to enhance transparency, it is not a mandatory requirement for scoping reviews. To promote transparency and reproducibility, we have clearly described our methodology in accordance with the Joanna Briggs Institute guidance and PRISMA-ScR reporting guidelines and have made all relevant data available through the supplementary material, as stated in the Data Availability Statement.

A two-pronged search strategy was applied, including a systematic literature search at the continental (Africa) level to determine research trends, and a targeted grey literature search at the regional level (SADC) to map climate related laws, policies, strategies, and plans relevant to health systems. This is a very long sentence and the intended meaning is not clear. Secondly, this sentence does not belong here, it should be put under search strategy.

Response: We agree and have restructured this sentence to improve clarity. The original formulation inadvertently conflated study design with search strategy. We have now clearly separated these elements, with the detailed search strategy described under "Information sources and search strategy." The revised text now reads: A two-pronged approach was applied to separately determine continental (Africa) research trends and regional (SADC) translation of global climate commitment into related laws, policies, strategies, and plans.

Accordingly, a systematic research literature search was conducted for the former and a targeted grey literature search for the latter.

The SADC - comprising 16 member states (Angola, Botswana, Comoros, Democratic Republic of the Congo, Eswatini, Lesotho, Madagascar, Malawi, Mauritius, Mozambique, Namibia, Seychelles, South Africa, Tanzania, Zambia, and Zimbabwe)-
The details of the specific countries should be moved to where the context of the review is being described.
Response: We agree and have moved the enumeration of the 16 SADC member states to the introduction where the study context is described.

Eligibility criteria: This section needs a complete revamp so that it is clearly stated for the readers to understand. Eligibility criteria is defined by inclusion and exclusion criteria. Thus, the eligibility criteria should be expressed in terms of Population, Concept and Context- PCC. This is better expressed in terms of the following table:
Table 1

	Inclusion criteria	Exclusion criteria
Population		
Concept		
Context		
Types of articles		

Response: Thank you for this suggestion. We agree that the eligibility criteria should be presented more clearly. We have substantially revised this section to address the reviewer's concern. We now explicitly frame our eligibility criteria according to the Population–Concept–Context (PCC) framework, which is the recommended approach for scoping reviews per Joanna Briggs Institute (JBI) guidance. The revised text clearly delineates:

- Population/Context: African health systems, as defined by WHO
- Concept: Climate change adaptation and mitigation within health systems
- Types of evidence: Research literature (primary research, reviews, editorials, commentaries, opinions, and conference abstracts) and grey literature (laws, policies, strategies, and plans)

- Exclusion criteria: Three distinct conditions are specified: i) sources addressing only health outcomes, ii) those excluding the health sector from climate action, and iii) superseded documents

Regarding presentation format, JBI guidance for scoping reviews recommends narrative description of PCC criteria rather than tabular presentation. Our revised narrative approach preserves clarity achieved through explicit PCC framing while maintaining manuscript flow. The revised text now reads: Eligibility criteria were developed according to the Population–Concept–Context (PCC) framework for scoping reviews. The population/context comprised African health systems, defined as "organisations, people and actions whose primary intent is to promote, restore or maintain health", while the concept was climate change adaptation and mitigation within health systems. Eligible sources included research literature (primary research, reviews, editorials, commentaries, opinions and conference abstracts) and grey literature (laws, policies, reports, strategies and plans). Sources were excluded if they focused solely on climate change-related health outcomes (e.g. climate-related disease mortality and morbidity), if climate change laws, policies, strategies and plans did not include the health sector, or if documents had been superseded by a newer version. No restrictions were applied by publication date or language.

Information source and search strategy: Although the search strategy was described, the process of selection is unclear. Meaning, a three-search strategy as defined by JBI scoping review methodology is not well elaborated. Kindly describe the 3-step search strategy so that this section becomes clear.

Response: We agree that the search strategy required further clarification. We have substantially revised this section to more clearly distinguish the search approaches used for the research literature and the grey literature (including targeted website searches), and to provide additional detail on how each search was conducted.

Information source and search strategy: The search included controlled and free text terms for synonyms of 'health care' or 'health facilities' and 'sustainable development' or 'low carbon' or 'climate change' or 'waste' and 'Africa'. The search words are not comprehensive. Words like climate change adaptation, mitigation, health systems, SADC and specific 16 countries within SADC should be the initial search words to be considered.

Response: We appreciate this suggestion. During development of the search strategy, we considered terms such as climate change adaptation, mitigation, and health systems. However, in consultation with the medical information specialist, we selected a combination of controlled vocabulary and synonymous free-text terms that were expected to maximise retrieval while limiting irrelevant records. For example, terms related to low carbon and environmental sustainability were used to capture mitigation, climate resilience to capture adaptation, and global warming to capture climate change. Country names for all SADC member states were also incorporated into the search strategy where relevant. The complete database-specific search strategies are provided in Supplemental material.

Information source and search strategy: The search was performed without restrictions for methodology, date or language. This has been said in one of the subheadings and why is it being repeated?
Response: We agree and have removed this repeated statement from the Information sources and search strategy section, as it is already described under Eligibility criteria.

Information source and search strategy: What was the role of the librarian in the search Strategy?
Response: The medical information specialist (JCFK), who is a co-author, contributed to the development and refinement of the search strategy, including the selection of controlled vocabulary and free-text search terms, optimisation of the database-specific search strategies, and deduplication of the search results.

Selection Process: Information on whether the duplicate were removed or not is not indicated in this section. Kindly indicate.
Response: Thank you for pointing this out. Duplicate records were removed before screening by the medical information specialist using EndNote, applying both the Amsterdam Efficient Deduplication ([16] DOI:10.5281/zenodo.3582927) and Bramer ([17] DOI:10.3163/1536-5050.104.3.014) methods, as described in the "Information sources and search strategy" section. We have retained this description in that section, as duplicate removal forms part of the search management process prior to study selection.

Selection process; What was the role of the librarian in this process

Response: The medical information specialist was not involved in the study selection process and contributed only to the development of the search strategy and management of the search results, including deduplication. Title/abstract screening and full-text screening were conducted by the review authors (MN and ÓBF), as described in the Selection process section.

Selection process: How was the reporting of the whole selection process done?
Response: The study selection process is reported in accordance with the PRISMA-ScR guidelines and illustrated in the PRISMA-ScR flow diagram (Figure 1), which details the identification, screening, eligibility assessment, inclusion of sources, and reasons for exclusion.

Data charting process: Rather call this subheading Data charting process and synthesis because this is what is indicated in the content
Response: We agree and have renamed the subheading to 'Data charting process and synthesis' to better reflect its contents.

Data charting process: For peer-reviewed literature, extracted data included study aims, objectives and key findings. Between the title and objective, may you please choose 1. Key findings should be tallied to answer the review questions.
Response: We agree that extracting both the title and objective was unnecessary. We have therefore retained only the study objectives, as these more clearly demonstrate how the included studies align with the review objectives and eligibility criteria. The extracted key findings are synthesised in the Results section according to the review questions.

RESULTS
The combined scientific and grey literature search yielded 15,365 records (Figure 1). Following title and abstract screening of peer-reviewed literature and executive summary screening of grey literature, 168 documents were assessed in full text. 85 peer reviewed publications and 15 grey literature documents were included, while 65 documents were excluded at the full-text stage. Kindly add a subheading for this content called Search results. While the authors tried to describe the results of the search process, there is need to include the reasons for exclusion at each stage of selection.

Response: We agree that this section benefits from a clearer introduction and have therefore added the subheading “Search results”. The reasons for exclusion at each stage of the selection process are reported in the PRISMA-ScR flow diagram (Figure 1), in accordance with PRISMA-ScR reporting guidance.

The included documents were published between 2006 and 2024, with most published from 2021 onwards. Studies were predominantly multinational (n=22), followed by country specific studies focused on South Africa (n=16), Egypt (n=12), and Nigeria (n=8). Evidence from other African countries, including Ghana, Kenya, Uganda, Zimbabwe, Morocco, Zambia, Zimbabwe, Chad, Somalia, Mozambique, Ethiopia, Benin, Algeria, Rwanda, and Senegal, was comparatively sparse, with four or fewer studies per country. Among peer reviewed publications, 62 reported original empirical studies, 22 were reviews, and two were editorials or perspectives. Kindly indicate the subheading for this content as publication characteristics

Response: We agree that a subheading is helpful to introduce the characteristics of the included evidence. We have therefore added the subheading “Literature characteristics” to this section.

The first part of results section was quantifying the results (for instance n=5) while the rest of the sections brought in references. There is need to maintain uniformity.

Response: The Results section was structured according to scoping review reporting conventions, with the “Search results” and “Literature characteristics” subsections providing a quantitative overview of the identified and included evidence, while the subsequent sections present a narrative synthesis of the findings supported by citations to the contributing studies. We agree that quantitative descriptions of the evidence base should also be consistently traceable to the underlying studies. We have therefore revised the Results section to include references [20-37], [38-53], [54-64], [65-70], [71-93] to the respective studies when reporting the number and characteristics of included studies, ensuring consistency in citation practices throughout the section.

DISCUSSION

At the continental level, we found limited and uneven mainstreaming of climate change into health professionals' education and training. Kindly reference the documents from

the identified studies talking to this.

Response: To support this interpretation, the paragraph now includes the relevant references [33,35,44] - DOI:10.7196/ajhpe.2023.v15i4.326; DOI:10.7196/AJHPE.2023.v15i1.1659, DOI:10.1016/j.afjem.2021.05.003.

Accordingly, Zolo (2024) and Al-Adawi (2022) propose the creation of knowledge platforms and the exchange of good practices among educators in institutions of higher learning to integrate ESH and PH into curricula and training. Intext referencing for the rest of the document is in terms of numbers. Kindly correct this.

Response: We have aligned the referencing style [23,119], as suggested.

Professional 'green teams' and individual champions have driven facility-level change, particularly in radiology, nursing and anaesthesiology, but their impact depends on leadership support and organisational mandate, underscoring the limits of individualled initiatives without system-level commitment. There is need to support this claim with a reference

Response: We agree and have added references [46,52,59] and [120] to the relevant included studies supporting this statement (DOI:10.7196/SAMJ.2017.v107i1.12023; DOI:10.1111/jan.16551; DOI: 10.4102/sajr.v28i1.3065 and DOI: 10.1016/j.healthpol.2024.105120).

African healthcare systems are among the most climate-vulnerable globally, as reflected in the predominant focus on adaptation. Across sub-Saharan Africa and SADC countries, responses prioritised emergency preparedness, disease surveillance and service delivery resilience to extreme weather events. This adaptation-led emphasis reflects rational prioritisation in fragile systems facing acute climate shocks, rather than policy neglect. That said, opportunities to anticipate and respond to climate-sensitive health risks such as heatwaves, vector-borne disease outbreaks, and extreme weather events remained under utilised. All these claims need to be referenced.

Response: We agree and have added references [92,93], [22,28,51,88,95], [45,51] to the relevant included studies supporting this statement (DOI:10.1080/14693062.2022.2098228 , DOI:10.1016/j.cliser.2023.100413, DOI: 10.5194/isprs-archives-XLVIII-2-2024-387-2024; DOI: 10.2471/BLT.23.020123; DOI: 10.4081/gh.2006.277; DOI: 10.1186/s12992-019-0466-x; DOI:10.11604/pamj.2021.40.113.30545; DOI: https://doi.org/10.1016/j.ijdrr.2017.10.012).

An assessment of SADC countries found uneven translation of international climate commitments into domestic law and policy. While the SADC climate change strategy and action plan provide a regional framework, only South Africa and Mauritius have enacted binding climate change legislation with explicit relevance to the health sector. Kindly reference these claims

Response: We agree and have added references [103,104] to the relevant included studies supporting this statement (links: SADC, Mauritius and South Africa).

Health National Adaptation Plans focus on vulnerability assessment and preparedness, while there is limited attention toward low-carbon and sustainable health system action planning. Kindly reference these claims

Response: We agree and have added a reference to support these statements (link: ATACH).

Overall, adaptation dominates research and policy responses, while healthcare system mitigation remains marginal. Acute climate shocks, fragile service capacity and competing health priorities incentivise reactive adaptation, while mitigation is deprioritised due to limited resources and the relatively low carbon footprint of African health systems. Kindly reference this claim

Response: We agree and have added references [129] and [130] to support these statements (Links: Lesotho and Ghanem).

Reviewer 2

Thank you for submitting this manuscript for review. Your topic is extremely relevant and addresses a major gap in our current knowledge and understanding of the climate impact of healthcare, particularly in Africa.

While your paper is strong, it does require some revisions to ensure reproducibility and the robustness of your conclusions. After a careful review of your paper, I have the following suggestions organized sequentially by section:

Methods

Page-6: your grey literature limited to SADC while your research literature includes all

Africa. This creates an asymmetry between policy and research results. This should be explained in your design choices.

Response: We agree and have expanded the methods section to explain and justify the two-pronged study design, including the rationale for examining research trends across Africa while focusing the policy analysis on the SADC region. The text now reads: A two-pronged approach was applied to separately determine continental (Africa) research trends and regional (SADC) translation of global climate commitments into related laws, policies, strategies, and plans. Accordingly, a systematic research literature search was conducted for the former and a targeted grey literature search for the latter. The SADC member states provide a relevant subregional case for examining gaps in translating global climate commitments into national health sector action. Member states face comparable climate-related risks but differ in health system capacity, governance arrangements and statutory maturity, enabling assessment of how similar global commitments are interpreted and operationalised across diverse national health system contexts.

Information Sources and Search Strategy

Page-7, line-58: "Documents were identified through hand-searching by country name, with titles and accompanying summaries screened within the first 10 pages of results." First 10 pages of results alone is not reproducible. Add the specific search engines/databases, search strings, and inclusion thresholds used.

Response: We agree that additional detail was needed to ensure reproducibility. We have expanded the description of the database and targeted website search strategies. The text now reads: Grey literature searches targeted climate change laws, policies, reports, strategies and plans produced by governments and key international organisations. Two complementary search strategies were employed: 1) a search of the Climate Change Laws of the World database using structured filters (country: SADC member states; document type: policy, law, UNFCCC submissions; sector: health), with screening of the first 200 results (approximately 10 pages) by title and summary; and 2) targeted website searches (Supplementary Table 1) of the SADC, country-specific national departments of health and environment, and the Alliance for Transformative Action on Climate and Health (ATACH) action progress tracker. The search result threshold (200 results and/or 10 pages) was pre-specified to balance comprehensiveness with feasibility, consistent with grey literature search protocols.

For website searches, country- and scope-specific search terms (country names and/or 'climate change') were used within each website's search function or document repository

when available. When a website search function was unavailable, the organisational structure was navigated manually to locate climate and health policy documents.

Selection Process

Page-8: "Title and abstract screening were conducted by one reviewer (MN), with a second reviewer (ÓBF)consulted in cases of uncertainty." This deviates from standard practice as usually dual independent screening is expected to avoid bias. This should be acknowledged in the paper or the language softened to account for this.

Response: We agree that the study selection process required further clarification. We have expanded the Methods section to explain that the eligibility criteria were first piloted independently by two reviewers to ensure consistent application before title and abstract screening proceeded. We also clarified that full-text screening was conducted independently by two reviewers. We have also added the deviation as a limitation in the Strengths and limitations section. The text reads: Title and abstract screening was primarily undertaken by one reviewer following pilot calibration with a second reviewer, which may have increased the risk of missed records.

Results

Page-9: In this section you note 15,365 records → 168 full-text → 85 + 15 included = 100; but when I look at Figure-1, the numbers do not align. The sum of records screened for both peer-reviewed and grey literature is 15,275. Total records included in the peer review is 90 in Figure-1. Please correct this discrepancy.

Response: Indeed, there were discrepancies between Figure 1 and the main text. We have amended both sections to be uniform. We have also reverted to the PRISMA flow diagram which does not include 'screened records' for literature obtained via other methods (grey literature). Therefore, the screened records (10,646) pertain only to the research literature.

Page-11, lines 3-7: you repeat public health expenditure modeling twice consecutively.

Response: The repeated line has been deleted.

References

I checked your citations and in your references you have two recorded under #25:

25. Irlam J, Razzack Z, Rother HA,...

25. Mohamed MAES, Ghallab E,...

I believe the latter should be #26 in your list of references. Scanning the text, I did not see any citations for #26. You'll likely need to double-check your citations under #25 and delineate those intended for #26 and update accordingly.
Response: The reference numbering has been corrected to be sequential. As the entire reference list has been revised, the associated numbers have changed to #43 and #63 for Irlam J, Razzack Z, Rother HA and Mohamed MAES, Ghallab, E, respectively. We have also reviewed the in-text citations and revised the relevant citations to ensure that references are correctly attributed and consistently cited throughout the manuscript.

You have duplicate references under #5 and #98 as the same paper appears under both.
Response: The duplicated reference #98 has been deleted and reference numbers adjusted accordingly.

For references #65 (HNAP) and Ref #72 (carbon footprint report), both point to the same ATACH URL (HNAP 2018). Please update #72 with a link to the correct document.
Response: The incorrect URL has been replaced with the correct link for reference #72 (now #100) (carbon footprint report) to the report.

You are missing a DOI for #35. The listed DOIs did not work for citations #43 and #54. For citation #84, the url does not work. A minor comment is that you are missing the "d" and just have "oi" in citation #39.
Response: The DOI have been added for references #35 (now #42 DOI:10.4102/SAJAA.v32i1.1506), #43 (now #87 https://doi.org/10.4102/phcfm.v14i1.3684) and #54 (now #61 DOI: 10.1016/j.aej.2020.08.011). The typographical error in reference #39 (now #51) has been corrected, and the URL for reference #84 (now #105) has been updated (Zambia).

You include some pre-prints under references #31 and #41. As these have not yet been peer-reviewed, this should be acknowledged.
Response: We have noted that the inclusion of pre-print references (now #75 and #20) was not explicitly stated and acknowledged the pre-prints in the subsection Literature characteristics in the Results section.

VERSION 2 - REVIEW

**Reviewer**	**2**
**Name**	**LaRoche, Jason**
**Affiliation**	**Janssen Research and Development LLC, Janssen Clinical Innovation**
**Date**	**25-Aug-2026**
**COI**	

Dear Dr. Netshiombo,

Thank you for revising your manuscript. It reads much more clearly than before. Aside from some spelling errors (see attached document), I found only a two minor errors that require correction:

Page-16, line-451: Check your units and/or decimal as the math is illogical. 68,603,660 tCO2e is more than twice the cited national emissions of 29,584,600 tCO2e.

References 30, 61, and 120 have incorrect DOIs. Please add the correct DOIs for these citations.

With these minor revisions, I believe that you have a very valuable manuscript that contributes to the scientific knowledge on the actions needed to adapt African healthcare systems for climate change.

Kind Regards,

Jason LaRoche

**** **The reviewer provided a marked copy with additional comments. Please contact the publisher for full details.** ****

VERSION 2 - AUTHOR RESPONSE

Reviewer 2

Thank you for revising your manuscript. It reads much more clearly than before. Aside from some spelling errors (see attached document), I found only a two minor errors that require correction:

Page-16, line-451: Check your units and/or decimal as the math is illogical. 68,603,660 tCO2e is more than twice the cited national emissions of 29,584,600 tCO2e.

References 30, 61, and 120 have incorrect DOIs. Please add the correct DOIs for these

citations.

With these minor revisions, I believe that you have a very valuable manuscript that contributes to the scientific knowledge on the actions needed to adapt African healthcare systems for climate change.

Add a decimal so this reads "10,646 records"

The record figure has been corrected to read 10,646.

Move the period to after the “t”

The punctuation has been corrected by moving the period to after the “t”.

Check your units and/or decimal. 68,603,660 tCO2e is more than twice the cited national emissions of 29,584,600 tCO2e.

Thank you for flagging this sentence, as the units could be misread. The health sector emissions figure is reported in tonnes of carbon dioxide equivalent (68,603.66 **t**CO2e) while the national figure is reported in kilotonnes of carbon dioxide equivalent (29,584.6 **kt**CO2e). To avoid misreading, we have clarified the units by spelling tCO2e (lines 348- 349) and ktCO2e (lines 446-447) in full.

Return the word "focus" to past tense (e.g. "focused").

The word has been corrected to the past tense “focused”.

DOI belongs to "An Interactive Sustainable Infrastructure Design Model for Health Clinics in Sub-Saharan Africa". Update to correct DOI.

We believed there might be a mismatch between the reference number cited in the reviewer’s introductory remarks (reference 30) and the reference highlighted in the reviewer’s PDF (reference 29). We have checked the highlighted reference (29) and confirm that the doi:10.1109/GHTC.2012.40 for “Sustainable Infrastructure Design Model for Health Clinics in Sub-Saharan Africa” is correct as originally cited. We reviewed reference 30; the DOI link was not opening, and we therefore updated it to doi:10.5334/aogh.4260.

DOI is incorrect. In earlier version below, it was correctly cited as 10.1016/j.aej.2020.08.011

We have corrected the DOI for reference 61 to doi:10.1016/j.aej.2020.08.011.

This DOI doesn't connect with the cited article

We have corrected the DOI for reference 120 to doi:10.1016/j.healthpol.2024.105120.

Thank you once again.
