# Supplementary material for: Climate change adaptation and mitigation in African health systems: a scoping review of research and policy translation
Source: BMJ Open. 2026 Sep 21;16(9):e119188. doi: 10.1136/bmjopen-2026-119188 (PMC13599907; doi:10.1136/bmjopen-2026-119188)
Supplement: Supplementary file 2 [file bmjopen-16-9-s001.docx]

**SUPPLEMENTAL FILE** **1**

**Full search strategies**

Search strategy for OVID/Medline (December 2, 2024)

*Ovid MEDLINE(R) ALL <1946 to November 27, 2024>*

| **1** | exp "Health Care Sector"/ or exp "Health Facilities"/ or (healthcare* or health-care* or health-facilit* or health-unit* or ((medical or clinical* or care or burn* or operat* or emergenc* or deliver*) adj3 (build* or unit* or room* or center* or centre*)) or rehabilitation-cent* or hospital or hospitals or clinic or clinics or ward or wards or ambulance* or nursing-home* or nursing-station* or (patient* adj3 room*)).ti,ab,kf. | **3334098** |
| --- | --- | --- |
| **2** | exp "Conservation of Natural Resources"/ or exp "Climate Change"/ or exp "Fossil Fuels"/ or exp "Medical Waste"/ or "Environmental Pollution"/ or "Air Pollution"/ or exp "Renewable Energy"/ or exp "Nuclear Energy"/ or (sustainab* or ((carbon* or nitrogen*) adj3 (emission* or pathway* or path-way* or foot-print* or footprint* or low* or zero)) or green-house or greenhouse or (climate* adj3 chang*) or global-warm* or zero-waste or fossil-fuel* or green-wash* or greenwash* or sdg or sdgs or smart-growth* or (conserv* adj3 natur*) or petrol* or gasoline or wastewater or waste-water or biowaste* or bio-waste* or ((medical* or pathological* or biologic* or hazard* or electronic* or solid or industr*) adj3 waste*) or ((water or air) adj3 polluti*) or green-health* or green-hospital* or diesel or green-energ* or windmill* or wind-mill* or windpower* or wind-power* or solar-energ* or solar-power* or solar-panel* or solar-energ* or geothermal-energ* or geo-thermal-energ* or nuclear-power* or nuclear-energ* or atomic-energ* or nuclear-react* or renewable* or water-power* or climate-impact* or climate-response* or climate-resilien* or sea-level-ris* or energy-transition* or energy-saving* or recycl* or reusab* or re-usab* or reuse* or circular* or biofuel* or bio-fuel* or coal).ti,ab,kf. | **946005** |
| **3** | exp "Africa"/ or (africa* or angola* or angolese* or angolian* or benin* or botswan* or burkina-faso* or burundi* or cabo-verde* or cameroon* or cape-verd* or chad or comoro* or congo* or cote-d-ivoire* or djibouti* or eritre* or eswatini* or ethiopia* or gabon* or gambia* or ghan* or (guinea* not (guinea-pig* or papua)) or ivory-coast* or kenya* or lesotho* or liberia* or madagascar* or malaw* or mali or malian or malians or mauritan* or mauriti* or mocambiqu* or mozambiqu* or namibia* or (niger* not aspergillus-niger) or rhodesia* or rwanda* or sao-tome* or senegal* or sierra-leone* or somalia* or sudan* or swaziland* or tanzan* or togo* or ugand* or zaire* or zambia* or zimbabw* or algeria* or morocc* or egypt* or libya* or tunisia*).ab,ti,kf. | **650995** |
| **4** | 1 and 2 and 3 | **5125** |

**Search strategy for Clarivate Analytics/Web of Science Core Collection (January 9, 2025)**

| **4** | #3 AND #2 AND #1 | **6,132** |
| --- | --- | --- |
| **3** | TS=(africa* OR angola* OR angolese* OR angolian* OR benin* OR botswan* OR burkina-faso* OR burundi* OR cabo-verde* OR cameroon* OR cape-verd* OR chad OR comoro* OR congo* OR cote-d-ivoire* OR djibouti* OR eritre* OR eswatini* OR ethiopia* OR gabon* OR gambia* OR ghan* OR ((guinea*) NOT (guinea-pig* OR papua)) OR ivory-coast* OR kenya* OR lesotho* OR liberia* OR madagascar* OR malaw* OR mali OR malian OR malians OR mauritan* OR mauriti* OR mocambiqu* OR mozambiqu* OR namibia* OR ((niger*) NOT (aspergillus-niger)) OR rhodesia* OR rwanda* OR sao-tome* OR senegal* OR sierra-leone* OR somalia* OR sudan* OR swaziland* OR tanzan* OR togo* OR ugand* OR zaire* OR zambia* OR zimbabw* OR algeria* OR morocc* OR egypt* OR libya* OR tunisia*) | **1,402,611** |
| **2** | TS=(sustainab* OR ((carbon* OR nitrogen*) NEAR/3 (emission* OR pathway* OR path-way* OR foot-print* OR footprint* OR low* OR zero)) OR green-house OR greenhouse OR (climate* NEAR/3 chang*) OR global-warm* OR zero-waste OR fossil-fuel* OR green-wash* OR greenwash* OR sdg OR sdgs OR smart-growth* OR (conserv* NEAR/3 natur*) OR petrol* OR gasoline OR wastewater OR waste-water OR biowaste* OR bio-waste* OR ((medical* OR pathological* OR biologic* OR hazard* OR electronic* OR solid OR industr*) NEAR/3 waste*) OR ((water OR air) NEAR/3 polluti*) OR green-health* OR green-hospital* OR diesel OR green-energ* OR windmill* OR wind-mill* OR windpower* OR wind-power* OR solar-energ* OR solar-power* OR solar-panel* OR solar-energ* OR geothermal-energ* OR geo-thermal-energ* OR nuclear-power* OR nuclear-energ* OR atomic-energ* OR nuclear-react* OR renewable* OR water-power* OR climate-impact* OR climate-response* OR climate-resilien* OR sea-level-ris* OR energy-transition* OR energy-saving* OR recycl* OR reusab* OR re-usab* OR reuse* OR circular* OR biofuel* OR bio-fuel* OR coal) | **3,409,084** |
| **1** | TS=(healthcare* OR health-care* OR health-facilit* OR health-unit* OR ((medical OR clinical* OR care OR burn* OR operat* OR emergenc* OR deliver*) NEAR/3 (build* OR unit* OR room* OR center* OR centre*)) OR rehabilitation-cent* OR hospital OR hospitals OR clinic OR clinics OR ward OR wards OR ambulance* OR nursing-home* OR nursing-station* OR (patient* NEAR/3 room*)) | **3,010,646** |

**Search strategy for Embase.com (January 9, 2025)**

| **No.** | **Query** | **Results** |
| --- | --- | --- |
| **#6** | #4 NOT #5 | **5489** |
| **#5** | #4 AND ('Conference Abstract'/it OR 'Conference Paper'/it) | **2061** |
| **#4** | #1 AND #2 AND #3 | **7550** |
| **#3** | 'africa'/exp OR africa*:ab,ti,kw OR angola*:ab,ti,kw OR angolese*:ab,ti,kw OR angolian*:ab,ti,kw OR benin*:ab,ti,kw OR botswan*:ab,ti,kw OR 'burkina faso*':ab,ti,kw OR burundi*:ab,ti,kw OR 'cabo verde*':ab,ti,kw OR cameroon*:ab,ti,kw OR 'cape verd*':ab,ti,kw OR chad:ab,ti,kw OR comoro*:ab,ti,kw OR congo*:ab,ti,kw OR 'cote d ivoire*':ab,ti,kw OR djibouti*:ab,ti,kw OR eritre*:ab,ti,kw OR eswatini*:ab,ti,kw OR ethiopia*:ab,ti,kw OR gabon*:ab,ti,kw OR gambia*:ab,ti,kw OR ghan*:ab,ti,kw OR (guinea*:ab,ti,kw NOT ('guinea pig*':ab,ti,kw OR papua:ab,ti,kw)) OR 'ivory coast*':ab,ti,kw OR kenya*:ab,ti,kw OR lesotho*:ab,ti,kw OR liberia*:ab,ti,kw OR madagascar*:ab,ti,kw OR malaw*:ab,ti,kw OR mali:ab,ti,kw OR malian:ab,ti,kw OR malians:ab,ti,kw OR mauritan*:ab,ti,kw OR mauriti*:ab,ti,kw OR mocambiqu*:ab,ti,kw OR mozambiqu*:ab,ti,kw OR namibia*:ab,ti,kw OR (niger*:ab,ti,kw NOT 'aspergillus niger':ab,ti,kw) OR rhodesia*:ab,ti,kw OR rwanda*:ab,ti,kw OR 'sao tome*':ab,ti,kw OR senegal*:ab,ti,kw OR 'sierra leone*':ab,ti,kw OR somalia*:ab,ti,kw OR sudan*:ab,ti,kw OR swaziland*:ab,ti,kw OR tanzan*:ab,ti,kw OR togo*:ab,ti,kw OR ugand*:ab,ti,kw OR zaire*:ab,ti,kw OR zambia*:ab,ti,kw OR zimbabw*:ab,ti,kw OR algeria*:ab,ti,kw OR morocc*:ab,ti,kw OR egypt*:ab,ti,kw OR libya*:ab,ti,kw OR tunisia*:ab,ti,kw | **813044** |
| **#2** | 'environmental protection'/de OR 'ecosystem restoration'/exp OR 'environmental sustainability'/exp OR 'environmentalism'/exp OR 'extended producer responsibility'/exp OR 'water conservation'/exp OR 'green chemistry'/exp OR 'climate change'/exp OR 'sustainable development'/exp OR 'fossil fuel'/exp OR 'hospital waste'/de OR 'hospital wastewater'/exp OR 'pollution'/de OR 'air pollution'/exp OR 'water pollution'/exp OR 'renewable energy'/exp OR 'renewable resource'/de OR 'nuclear energy and related phenomena'/exp OR 'emission'/exp OR 'nitrogen deposition'/exp OR 'global warming potential'/exp OR 'biowaste'/exp OR 'wind farm'/exp OR 'geothermal power plant'/exp OR 'hydroelectric power plant'/exp OR 'nuclear power plant'/exp OR 'solar power plant'/exp OR 'sea level rise'/exp OR 'human impact (environment)'/exp OR 'energy conservation'/exp OR sustainab*:ti,ab,kw OR (((carbon* OR nitrogen*) NEAR/3 (emission* OR pathway* OR 'path way*' OR 'foot print*' OR footprint* OR low* OR zero)):ti,ab,kw) OR 'green house':ti,ab,kw OR greenhouse:ti,ab,kw OR ((climate* NEAR/3 chang*):ti,ab,kw) OR 'global warm*':ti,ab,kw OR 'zero waste':ti,ab,kw OR 'fossil fuel*':ti,ab,kw OR 'green wash*':ti,ab,kw OR greenwash*:ti,ab,kw OR sdg:ti,ab,kw OR sdgs:ti,ab,kw OR 'smart growth*':ti,ab,kw OR ((conserv* NEAR/3 natur*):ti,ab,kw) OR petrol*:ti,ab,kw OR gasoline:ti,ab,kw OR wastewater:ti,ab,kw OR 'waste water':ti,ab,kw OR biowaste*:ti,ab,kw OR 'bio waste*':ti,ab,kw OR (((medical* OR pathological* OR biologic* OR hazard* OR electronic* OR solid OR industr*) NEAR/3 waste*):ti,ab,kw) OR (((water OR air) NEAR/3 polluti*):ti,ab,kw) OR 'green health*':ti,ab,kw OR 'green hospital*':ti,ab,kw OR diesel:ti,ab,kw OR 'green energ*':ti,ab,kw OR windmill*:ti,ab,kw OR 'wind mill*':ti,ab,kw OR windpower*:ti,ab,kw OR 'wind power*':ti,ab,kw OR 'solar power*':ti,ab,kw OR 'solar panel*':ti,ab,kw OR 'solar energ*':ti,ab,kw OR 'geothermal energ*':ti,ab,kw OR 'geo thermal energ*':ti,ab,kw OR 'nuclear power*':ti,ab,kw OR 'nuclear energ*':ti,ab,kw OR 'atomic energ*':ti,ab,kw OR 'nuclear react*':ti,ab,kw OR renewable*:ti,ab,kw OR 'water power*':ti,ab,kw OR 'climate impact*':ti,ab,kw OR 'climate response*':ti,ab,kw OR 'climate resilien*':ti,ab,kw OR 'sea level ris*':ti,ab,kw OR 'energy transition*':ti,ab,kw OR 'energy saving*':ti,ab,kw OR recycl*:ti,ab,kw OR reusab*:ti,ab,kw OR 're usab*':ti,ab,kw OR reuse*:ti,ab,kw OR circular*:ti,ab,kw OR biofuel*:ti,ab,kw OR 'bio fuel*':ti,ab,kw OR coal:ti,ab,kw | **1270021** |
| **#1** | 'health care facilities and services'/de OR 'health care facility'/exp OR 'nursing station'/exp OR healthcare*:ti,ab,kw OR 'health care*':ti,ab,kw OR 'health facilit*':ti,ab,kw OR 'health unit*':ti,ab,kw OR (((medical OR clinical* OR care OR burn* OR operat* OR emergenc* OR deliver*) NEAR/3 (build* OR unit* OR room* OR center* OR centre*)):ti,ab,kw) OR 'rehabilitation cent*':ti,ab,kw OR hospital:ti,ab,kw OR hospitals:ti,ab,kw OR clinic:ti,ab,kw OR clinics:ti,ab,kw OR ward:ti,ab,kw OR wards:ti,ab,kw OR ambulance*:ti,ab,kw OR 'nursing home*':ti,ab,kw OR 'nursing station*':ti,ab,kw OR ((patient* NEAR/3 room*):ti,ab,kw) | **5220497** |

**Search strategy for Ebsco/CINAHL Plus With Fulltext (January 9, 2025)**

| **#** | **Query** | **Results** |
| --- | --- | --- |
| **S4** | S1 AND S2 AND S3 | **2,080** |
| **S3** | (MH "Africa+") OR (MH "Africa, Northern+") OR (MH "Africa South of the Sahara+") OR (MH "Africa, Central+") OR (MH "Africa, Eastern+") OR (MH "Africa, Southern+") OR (MH "Africa, Western+") OR TI(africa* OR angola* OR angolese* OR angolian* OR benin* OR botswan* OR burkina-faso* OR burundi* OR cabo-verde* OR cameroon* OR cape-verd* OR chad OR comoro* OR congo* OR cote-d-ivoire* OR djibouti* OR eritre* OR eswatini* OR ethiopia* OR gabon* OR gambia* OR ghan* OR ((guinea*) NOT (guinea-pig* OR papua)) OR ivory-coast* OR kenya* OR lesotho* OR liberia* OR madagascar* OR malaw* OR mali OR malian OR malians OR mauritan* OR mauriti* OR mocambiqu* OR mozambiqu* OR namibia* OR ((niger*) NOT (aspergillus-niger)) OR rhodesia* OR rwanda* OR sao-tome* OR senegal* OR sierra-leone* OR somalia* OR sudan* OR swaziland* OR tanzan* OR togo* OR ugand* OR zaire* OR zambia* OR zimbabw* OR algeria* OR morocc* OR egypt* OR libya* OR tunisia*) OR AB(africa* OR angola* OR angolese* OR angolian* OR benin* OR botswan* OR burkina-faso* OR burundi* OR cabo-verde* OR cameroon* OR cape-verd* OR chad OR comoro* OR congo* OR cote-d-ivoire* OR djibouti* OR eritre* OR eswatini* OR ethiopia* OR gabon* OR gambia* OR ghan* OR ((guinea*) NOT (guinea-pig* OR papua)) OR ivory-coast* OR kenya* OR lesotho* OR liberia* OR madagascar* OR malaw* OR mali OR malian OR malians OR mauritan* OR mauriti* OR mocambiqu* OR mozambiqu* OR namibia* OR ((niger*) NOT (aspergillus-niger)) OR rhodesia* OR rwanda* OR sao-tome* OR senegal* OR sierra-leone* OR somalia* OR sudan* OR swaziland* OR tanzan* OR togo* OR ugand* OR zaire* OR zambia* OR zimbabw* OR algeria* OR morocc* OR egypt* OR libya* OR tunisia*) OR SU(africa* OR angola* OR angolese* OR angolian* OR benin* OR botswan* OR burkina-faso* OR burundi* OR cabo-verde* OR cameroon* OR cape-verd* OR chad OR comoro* OR congo* OR cote-d-ivoire* OR djibouti* OR eritre* OR eswatini* OR ethiopia* OR gabon* OR gambia* OR ghan* OR ((guinea*) NOT (guinea-pig* OR papua)) OR ivory-coast* OR kenya* OR lesotho* OR liberia* OR madagascar* OR malaw* OR mali OR malian OR malians OR mauritan* OR mauriti* OR mocambiqu* OR mozambiqu* OR namibia* OR ((niger*) NOT (aspergillus-niger)) OR rhodesia* OR rwanda* OR sao-tome* OR senegal* OR sierra-leone* OR somalia* OR sudan* OR swaziland* OR tanzan* OR togo* OR ugand* OR zaire* OR zambia* OR zimbabw* OR algeria* OR morocc* OR egypt* OR libya* OR tunisia*) | **182,453** |
| **S2** | (MH "Sustainable Growth") OR (MH "Environmental Pollutants+") OR (MH "Air Pollutants+") OR (MH "Environmental Sustainability") OR (MH "Carbon Footprint") OR (MH "Conservation of Natural Resources+") OR (MH "Fossil Fuels+") OR (MH "Nuclear Energy") OR (MH "Sea Level Rise") OR (MH "Ecosystem") OR (MH "Energy-Generating Resources") OR (MH "Medical Waste Disposal") OR (MH "Medical Waste") OR (MH "Environmental Pollution") OR (MH "Climate Change") OR (MH "Greenhouse Effect") OR (MH "Nuclear Energy") OR (MH "Nuclear Reactors") OR (MH "Air Pollution") OR (MH "Motor Vehicle Emissions") OR (MH "Traffic Pollution") OR (MH "Sea Level Rise") OR TI(sustainab* OR ((carbon* OR nitrogen*) N3 (emission* OR pathway* OR path-way* OR foot-print* OR footprint* OR low* OR zero)) OR green-house OR greenhouse OR (climate* N3 chang*) OR global-warm* OR zero-waste OR fossil-fuel* OR green-wash* OR greenwash* OR sdg OR sdgs OR smart-growth* OR (conserv* N3 natur*) OR petrol* OR gasoline OR wastewater OR waste-water OR biowaste* OR bio-waste* OR ((medical* OR pathological* OR biologic* OR hazard* OR electronic* OR solid OR industr*) N3 waste*) OR ((water OR air) N3 polluti*) OR green-health* OR green-hospital* OR diesel OR green-energ* OR windmill* OR wind-mill* OR windpower* OR wind-power* OR solar-energ* OR solar-power* OR solar-panel* OR solar-energ* OR geothermal-energ* OR geo-thermal-energ* OR nuclear-power* OR nuclear-energ* OR atomic-energ* OR nuclear-react* OR renewable* OR water-power* OR climate-impact* OR climate-response* OR climate-resilien* OR sea-level-ris* OR energy-transition* OR energy-saving* OR recycl* OR reusab* OR re-usab* OR reuse* OR circular* OR biofuel* OR bio-fuel* OR coal) OR AB(sustainab* OR ((carbon* OR nitrogen*) N3 (emission* OR pathway* OR path-way* OR foot-print* OR footprint* OR low* OR zero)) OR green-house OR greenhouse OR (climate* N3 chang*) OR global-warm* OR zero-waste OR fossil-fuel* OR green-wash* OR greenwash* OR sdg OR sdgs OR smart-growth* OR (conserv* N3 natur*) OR petrol* OR gasoline OR wastewater OR waste-water OR biowaste* OR bio-waste* OR ((medical* OR pathological* OR biologic* OR hazard* OR electronic* OR solid OR industr*) N3 waste*) OR ((water OR air) N3 polluti*) OR green-health* OR green-hospital* OR diesel OR green-energ* OR windmill* OR wind-mill* OR windpower* OR wind-power* OR solar-energ* OR solar-power* OR solar-panel* OR solar-energ* OR geothermal-energ* OR geo-thermal-energ* OR nuclear-power* OR nuclear-energ* OR atomic-energ* OR nuclear-react* OR renewable* OR water-power* OR climate-impact* OR climate-response* OR climate-resilien* OR sea-level-ris* OR energy-transition* OR energy-saving* OR recycl* OR reusab* OR re-usab* OR reuse* OR circular* OR biofuel* OR bio-fuel* OR coal) OR SU(sustainab* OR ((carbon* OR nitrogen*) N3 (emission* OR pathway* OR path-way* OR foot-print* OR footprint* OR low* OR zero)) OR green-house OR greenhouse OR (climate* N3 chang*) OR global-warm* OR zero-waste OR fossil-fuel* OR green-wash* OR greenwash* OR sdg OR sdgs OR smart-growth* OR (conserv* N3 natur*) OR petrol* OR gasoline OR wastewater OR waste-water OR biowaste* OR bio-waste* OR ((medical* OR pathological* OR biologic* OR hazard* OR electronic* OR solid OR industr*) N3 waste*) OR ((water OR air) N3 polluti*) OR green-health* OR green-hospital* OR diesel OR green-energ* OR windmill* OR wind-mill* OR windpower* OR wind-power* OR solar-energ* OR solar-power* OR solar-panel* OR solar-energ* OR geothermal-energ* OR geo-thermal-energ* OR nuclear-power* OR nuclear-energ* OR atomic-energ* OR nuclear-react* OR renewable* OR water-power* OR climate-impact* OR climate-response* OR climate-resilien* OR sea-level-ris* OR energy-transition* OR energy-saving* OR recycl* OR reusab* OR re-usab* OR reuse* OR circular* OR biofuel* OR bio-fuel* OR coal) | **92,898** |
| **S1** | (MH "Health Facilities+") OR (MH "Community Health Centers+") OR (MH "Health Facility Departments+") OR (MH "Clinical Laboratories+") OR (MH "Emergency Service+") OR (MH "Midwifery Service+") OR (MH "Pharmacy Service+") OR (MH "Hospital Units+") OR (MH "Delivery Rooms+") OR (MH "Intensive Care Units+") OR (MH "Pediatric Units+") OR (MH "Rehabilitation Centers+") OR (MH "Hospitals+") OR (MH "Hospitals, Public+") OR (MH "Hospitals, Special+") OR (MH "Patients' Rooms+") OR (MH "Nursing Homes+") OR (MH "Residential Facilities+") OR TI(healthcare* OR health-care* OR health-facilit* OR health-unit* OR ((medical OR clinical* OR care OR burn* OR operat* OR emergenc* OR deliver*) N3 (build* OR unit* OR room* OR center* OR centre*)) OR rehabilitation-cent* OR hospital OR hospitals OR clinic OR clinics OR ward OR wards OR ambulance* OR nursing-home* OR nursing-station* OR (patient* N3 room*)) OR AB(healthcare* OR health-care* OR health-facilit* OR health-unit* OR ((medical OR clinical* OR care OR burn* OR operat* OR emergenc* OR deliver*) N3 (build* OR unit* OR room* OR center* OR centre*)) OR rehabilitation-cent* OR hospital OR hospitals OR clinic OR clinics OR ward OR wards OR ambulance* OR nursing-home* OR nursing-station* OR (patient* N3 room*)) OR SU(healthcare* OR health-care* OR health-facilit* OR health-unit* OR ((medical OR clinical* OR care OR burn* OR operat* OR emergenc* OR deliver*) N3 (build* OR unit* OR room* OR center* OR centre*)) OR rehabilitation-cent* OR hospital OR hospitals OR clinic OR clinics OR ward OR wards OR ambulance* OR nursing-home* OR nursing-station* OR (patient* N3 room*)) | **1,724,035** |

**Search strategy for Elsevier/Scopus (January 10, 2025)**

| **26** | #23 AND #24 AND #25 | **7,285** |
| --- | --- | --- |
| **25** | TITLE-ABS (africa* OR angola* OR angolese* OR angolian* OR benin* OR botswan* OR burkina-faso* OR burundi* OR cabo-verde* OR cameroon* OR cape-verd* OR chad OR comoro* OR congo* OR cote-d-ivoire* OR djibouti* OR eritre* OR eswatini* OR ethiopia* OR gabon* OR gambia* OR ghan* OR ((guinea*) AND NOT (guinea-pig* OR papua)) OR ivory-coast* OR kenya* OR lesotho* OR liberia* OR madagascar* OR malaw* OR mali OR malian OR malians OR mauritan* OR mauriti* OR mocambiqu* OR mozambiqu* OR namibia* OR ((niger*) AND NOT (aspergillus-niger)) OR rhodesia* OR rwanda* OR sao-tome* OR senegal* OR sierra-leone* OR somalia* OR sudan* OR swaziland* OR tanzan* OR togo* OR ugand* OR zaire* OR zambia* OR zimbabw* OR algeria* OR morocc* OR egypt* OR libya* OR tunisia*) OR AUTHKEY (africa* OR angola* OR angolese* OR angolian* OR benin* OR botswan* OR burkina-faso* OR burundi* OR cabo-verde* OR cameroon* OR cape-verd* OR chad OR comoro* OR congo* OR cote-d-ivoire* OR djibouti* OR eritre* OR eswatini* OR ethiopia* OR gabon* OR gambia* OR ghan* OR ((guinea*) AND NOT (guinea-pig* OR papua)) OR ivory-coast* OR kenya* OR lesotho* OR liberia* OR madagascar* OR malaw* OR mali OR malian OR malians OR mauritan* OR mauriti* OR mocambiqu* OR mozambiqu* OR namibia* OR ((niger*) AND NOT (aspergillus-niger)) OR rhodesia* OR rwanda* OR sao-tome* OR senegal* OR sierra-leone* OR somalia* OR sudan* OR swaziland* OR tanzan* OR togo* OR ugand* OR zaire* OR zambia* OR zimbabw* OR algeria* OR morocc* OR egypt* OR libya* OR tunisia*) | **1,580,728** |
| **24** | TITLE-ABS (sustainab* OR ((carbon* OR nitrogen*) W/3 (emission* OR pathway* OR path-way* OR foot-print* OR footprint* OR low* OR zero)) OR green-house OR greenhouse OR (climate* W/3 chang*) OR global-warm* OR zero-waste OR fossil-fuel* OR green-wash* OR greenwash* OR sdg OR sdgs OR smart-growth* OR (conserv* W/3 natur*) OR petrol* OR gasoline OR wastewater OR waste-water OR biowaste* OR bio-waste* OR ((medical* OR pathological* OR biologic* OR hazard* OR electronic* OR solid OR industr*) W/3 waste*) OR ((water OR air) W/3 polluti*) OR green-health* OR green-hospital* OR diesel OR green-energ* OR windmill* OR wind-mill* OR windpower* OR wind-power* OR solar-energ* OR solar-power* OR solar-panel* OR solar-energ* OR geothermal-energ* OR geo-thermal-energ* OR nuclear-power* OR nuclear-energ* OR atomic-energ* OR nuclear-react* OR renewable* OR water-power* OR climate-impact* OR climate-response* OR climate-resilien* OR sea-level-ris* OR energy-transition* OR energy-saving* OR recycl* OR reusab* OR re-usab* OR reuse* OR circular* OR biofuel* OR bio-fuel* OR coal) OR AUTHKEY (sustainab* OR ((carbon* OR nitrogen*) W/3 (emission* OR pathway* OR path-way* OR foot-print* OR footprint* OR low* OR zero)) OR green-house OR greenhouse OR (climate* W/3 chang*) OR global-warm* OR zero-waste OR fossil-fuel* OR green-wash* OR greenwash* OR sdg OR sdgs OR smart-growth* OR (conserv* W/3 natur*) OR petrol* OR gasoline OR wastewater OR waste-water OR biowaste* OR bio-waste* OR ((medical* OR pathological* OR biologic* OR hazard* OR electronic* OR solid OR industr*) W/3 waste*) OR ((water OR air) W/3 polluti*) OR green-health* OR green-hospital* OR diesel OR green-energ* OR windmill* OR wind-mill* OR windpower* OR wind-power* OR solar-energ* OR solar-power* OR solar-panel* OR solar-energ* OR geothermal-energ* OR geo-thermal-energ* OR nuclear-power* OR nuclear-energ* OR atomic-energ* OR nuclear-react* OR renewable* OR water-power* OR climate-impact* OR climate-response* OR climate-resilien* OR sea-level-ris* OR energy-transition* OR energy-saving* OR recycl* OR reusab* OR re-usab* OR reuse* OR circular* OR biofuel* OR bio-fuel* OR coal) | **5,001,799** |
| **23** | TITLE-ABS (healthcare* OR health-care* OR health-facilit* OR health-unit* OR ((medical OR clinical* OR care OR burn* OR operat* OR emergenc* OR deliver*) W/3 (build* OR unit* OR room* OR center* OR centre*)) OR rehabilitation-cent* OR hospital OR hospitals OR clinic OR clinics OR ward OR wards OR ambulance* OR nursing-home* OR nursing-station* OR (patient* W/3 room*)) OR AUTHKEY (healthcare* OR health-care* OR health-facilit* OR health-unit* OR ((medical OR clinical* OR care OR burn* OR operat* OR emergenc* OR deliver*) W/3 (build* OR unit* OR room* OR center* OR centre*)) OR rehabilitation-cent* OR hospital OR hospitals OR clinic OR clinics OR ward OR wards OR ambulance* OR nursing-home* OR nursing-station* OR (patient* W/3 room*)) | **4,047,648** |

**Search strategy for Global Health Index/African Index Medicus (January 10, 2025)**

1. **Health care sector, health care services, health care system**

(healthcare* OR health-care* OR health-facilit* OR health-unit* OR ((medical OR clinical* OR care OR burn* OR operat* OR emergenc* OR deliver*) AND (build* OR unit* OR room* OR center* OR centre*)) OR rehabilitation-cent* OR hospital OR hospitals OR clinic OR clinics OR ward OR wards OR ambulance* OR nursing-home* OR nursing-station* OR (patient* AND room*))

2. **Sustainable development, low carbon pathways, green healthcare systems, Carbon emmissions, green house gases, carbon footprint, climate change, medical waste, pollution**

(sustainab* OR ((carbon* OR nitrogen*) AND (emission* OR pathway* OR path-way* OR foot-print* OR footprint* OR low* OR zero)) OR green-house OR greenhouse OR (climate* AND chang*) OR global-warm* OR zero-waste OR fossil-fuel* OR green-wash* OR greenwash* OR sdg OR sdgs OR smart-growth* OR (conserv* AND natur*) OR petrol* OR gasoline OR wastewater OR waste-water OR biowaste* OR bio-waste* OR ((medical* OR pathological* OR biologic* OR hazard* OR electronic* OR solid OR industr*) AND waste*) OR ((water OR air) AND polluti*) OR green-health* OR green-hospital* OR diesel OR green-energ* OR windmill* OR wind-mill* OR windpower* OR wind-power* OR solar-energ* OR solar-power* OR solar-panel* OR solar-energ* OR geothermal-energ* OR geo-thermal-energ* OR nuclear-power* OR nuclear-energ* OR atomic-energ* OR nuclear-react* OR renewable* OR water-power* OR climate-impact* OR climate-response* OR climate-resilien* OR sea-level-ris* OR energy-transition* OR energy-saving* OR recycl* OR reusab* OR re-usab* OR reuse* OR circular* OR biofuel* OR bio-fuel* OR coal)

3. **Africa (all 54 countries)**
