# Supplementary material for: Climate change adaptation and mitigation in African health systems: a scoping review of research and policy translation
Source: BMJ Open. 2026 Sep 21;16(9):e119188. doi: 10.1136/bmjopen-2026-119188 (PMC13599907; doi:10.1136/bmjopen-2026-119188)
Supplement: Supplementary file 3 [file bmjopen-16-9-s002.docx]

**Supplemental Table 1: Targeted websites and organisations searched**

| **#** | **Website name/organization** | **Link** |
| --- | --- | --- |
| 1 | South African Development Community | https://www.sadc.int |
| 2 | ATACH Community | https://www.atachcommunity.com |
| 3 | Climate Change Laws of the World | https://climate-laws.org |
| 4 | Ministry of Health (Angola) | https://www.minsa.gov.ao |
| 5 | Ministry of Health and Wellness (Botswana) | https://www.moh.gov.bw |
| 6 | Ministry of Public Health and Population (Comoros) | https://www.mspp.gouv.ht |
| 7 | Ministry of Public Health, Hygiene and Social Welfare (Democratic Republic of Congo) | https://sante.gouv.cd |
| 8 | Ministry of Health (Kingdon of Lesotho) | https://health.gov.ls |
| 9 | Ministry of Public Health (Madagascar) | https://www.msanp.gov.mg |
| 10 | Ministry of Health and Sanitation (Malawi) | https://www.health.gov.mw |
| 11 | Ministry of Health and Wellness (Mauritius) | https://health.govmu.org/health/ |
| 12 | Department of Health (South Africa) | https://www.health.gov.za |
| 13 | Ministry of Health (United Republic of Tanzania) | https://www.moh.go.tz |
| 14 | Ministry of Health (Zambia) | https://www.moh.gov.zm |
| 15 | Ministry of Health and Child Care (Zimbabwe) | https://www.mohcc.gov.zw |
| 16 | Ministry of the Environment (Angola) | https://www.minamb.gov.ao |
| 17 | Ministry of Health and Tourism (Botswana) | https://www.gov.bw/ministries/ministry-environment-and-tourism |
| 18 | Ministry of Ministry of Environment, Sustainable Development, and Climate Economy (Democratic Republic of Congo) | https://medd.gouv.cd |
| 19 | Department of Environment (Kingdon of Lesotho) | https://environment.gov.ls |
| 20 | Ministry of Environment and Sustainable Development (Madagascar) | https://www.environnement.mg |
| 21 | Ministry of Natural Resources (Malawi) | https://mnr.gov.mw |
| 22 | Ministry of Environment, Solid Waste Management and Climate Change (Mauritius) | https://environment.govmu.org/Pages/Index.aspx |
| 23 | Ministry of Environment, Forestry and Tourism (Namibia) | https://www.meft.gov.na |
| 24 | Department of Forestry, Fisheries and the Environment (South Africa) | https://www.dffe.gov.za |
| 25 | Vice President’s Office (United Republic of Tanzania) | https://www.vpo.go.tz |
| 26 | Ministry of Green Economy and Environment (Zambia) | https://www.mgee.gov.zm |
