# Supplementary material for: Climate change adaptation and mitigation in African health systems: a scoping review of research and policy translation
Source: BMJ Open. 2026 Sep 21;16(9):e119188. doi: 10.1136/bmjopen-2026-119188 (PMC13599907; doi:10.1136/bmjopen-2026-119188)
Supplement: Supplementary file 4 [file bmjopen-16-9-s003.docx]

**Supplemental Table 2: Characteristics of the included research publications**

| **Country** | **Author/Year** | **Objective** | **Study Design** | **Key findings** | **WHO Domains** | **Adaptation or Mitigation**  **focus** |
| --- | --- | --- | --- | --- | --- | --- |
| Algeria | Mounir, Rima and Aziz, 2013 | To determine the technical&economic feasibility of solar water heating integration in hospital centres. | Modelling | Solar water heating reduced emissions by 79.8 tCO₂, contributing to energy savings and cost efficiency. Nationally equates to a 0.02% emission reduction. | Climate-Resilient and Sustainable Technologies and Infrastructure and Supply Chain | **Mitigation** |
| Benin | Singh et al | To explores the use of machine learning techniques to predict malaria outbreaks. | Modelling | Machine learning showed potential in predicting potential malaria outbreaks in high risk zones during seasonal rainfall. | Integrated Risk Monitoring and Early Warning, and GHG Emission Tracking | **Adaptation** |
| Chad | Allarané et al, 2024 | To investigate climate risks and adaptation strategies used to support climate shocks | Interviews, Documentary | Climate shocks cause infrastructural degradation, disruption of services&increased energy consumption. | Climate Smart Health Workforce | **Adaptation** |
| Egypt | Moustafa Saleh and lsabahy, 2022 | To determine nursing interns’ attitudes related to sustainable development in health care | Cross-sectional | Most interns had little knowledge of environmental sustainability. Education on sustainable healthcare led to significantly improved attitude, knowledge and behaviour toward ES. | Climate Smart Health Workforce | **Adaptation** |
|  | William et al, 2020 | To assess the energy efficiency and environmental performance of a hospital building | Modelling | Energy efficient-HVAC systems coupled with sustainable energy use can reduce carbon emissions by 12% - 67%. | Climate resilient and low carbon infrastructure, technologies, and supply chain | **Mitigation** |
|  | Atia, Bassily and Elame, 2020 | To examine whether the LCC and LCA support sustainable decision making in the health sector. | Mixed methods | Integrating LCC and LCA supports decision-making that contributes to cost, environmental and economic efficient co-benefits. | Climate-Informed Health Research | **Adaptation** |
|  | Momen, 2024 | To evaluate the perception of medical students towards climate change. | Cross sectional | Half of the students believe climate change should be integrated into medical curriculum. 21.5% reported being educated on climate change in medical school. | Climate Smart Health Workforce | **Adaptation** |
|  | Momen, Allam and Basyoni, 2024 | To determine family medicine physician attitudes towards climate change | Cross sectional | 9.6% of physicians feel adequately informed about climate change and health and less than 10% discuss climate change with their patients. | Climate Smart Health Workforce | **Adaptation** |
|  | Mousa, Puhakka and Abdelgaffar, 2019 | To explore the relationship between climate change and the organisational commitment | Cross sectional | Leadership had a negligible impact on the relationship between climate change and commitments. Hospitals pay minimal attention to climate change impacts, which undermines employee commitment. | Climate Smart Health Workforce | **Adaptation** |
|  | Mohammed, 2024 | Evaluate the effect of a nurse-led climate change educational program for asthmatic women | Cross sectional | The educational program positively impacted women's' knowledge and reported practices concerning climate change, in relation to their asthma management. | Climate-Informed Health Programs | **Adaptation** |
|  | Abdelaziz, 2024 | To compare nurses’ knowledge, skills, and attitudes regarding climate change. | Cross sectional | In Beni-Suef hospitals, a significant number of participants exhibited poor knowledge, skills, and attitudes (48,2 %, 50,6 %, and 54,2 %, respectively) toward climate change compared to those in Al Azhar (69,6 %, 29,2 %, and 82,1 %). | Climate Smart Health Workforce | **Adaptation** |
|  | El-Sayed, 2024 | To explore organisational agility, climate activism, and green work behaviour among nurses. | Cross sectional | Organisational agility was strongly linked to climate activism and green work behaviour. | Climate Smart Health Workforce | **Adaptation** |
|  | Atta et al, 2024 | To identify the predictors of climate change literacy among nursing students. | Cross sectional | Perceived faculty staff’s knowledge of climate change had influence on student’s climate change literacy. | Climate Smart Health Workforce | **Adaptation** |
|  | Mohamed et al, 2024 | To assess sustainability consciousness among nursing students. | Cross sectional | Within the knowledge construct, the environmental dimension scored 2nd highest mean score, while it scored lowest within the attitudes construct, and highest in the sustainability behaviour construct. | Climate Smart Health Workforce | **Adaptation** |
| Ethiopia | Ngusie and Rufo, 2024 | To explore the technical&economic feasibility of renewable energy use in a health centre. | Modelling | Reduction of GHG emissions has a monetary value: eliminating diesel systems for hybrid PV battery energy can save $62 084.75 per annum. | Climate-Resilient and Sustainable Technologies and Infrastructure | **Mitigation** |
| Ghana | Guo et al, 2020 | To evaluate the green procurement practices in mining hospitals. | Interviews | Green procurement practices were limited and inconsistently applied –Lack of awareness and weak regulatory enforcement affected practices. | Climate-Resilient and Sustainable Technologies and Infrastructure and Supply Chain | **Adaptation** |
|  | Barimah et al, 2023 | To investigate health professionals’ understanding of the effects of climate change on health. | Cross-sectional (pre-print) | Majority of participants believe there is a between climate change and health including impact on children and mental health. Majority supported mitigation efforts: reducing energy use, adopting renewable energy, and promoting public transport use or cycling. | Climate Smart Health Workforce | **Adaptation** |
|  | Codjoe et al, 2020 | To explore the vulnerabilities of health services during flooding and extreme heat in two urban areas. | Mixed methods | Floods damage equipment, disrupt power supply and block access roads. Heat increases poor ventilation, high indoor temperatures, and causes hygiene disruptions. | Climate and Health Risk and GHG Emissions Assessment | **Adaptation** |
|  | Damoah, Ayakwah and Tingbani, 2021 | To determine whether medical drones impact the socio-economic and environmental SDGs. | Interviews and Documentary | Use of battery run drones in health system supply chain instead of cars and motorcycles reduces carbon emissions. | Climate Smart Health Workforce | **Mitigation** |
| Kenya | Yakub, Shah and Sokhi, 2023 | To measure the environmental impact of delivering care through tele-neurology consultations. | Cross-sectional | The use of tele-consultations over in-person consultations resulted 3.5t of CO2 emission reduction. | Climate Smart Health Workforce | **Mitigation** |
|  | Sheriff and Mash, 2022 | To explore the impact of climate change on PHC. | Report | Lack of water and high temperatures negatively impact the resilience of primary care and services. | Climate and Health Risk and GHG Emissions Assessment | **Adaptation** |
|  | Andersen, et al, 2021 | To explore the knowledge on climate change related health risks and adaptation strategies. | Interviews | Weaknesses in urban planning made healthcare facilities vulnerable to infrastructural damage during floods, affecting service delivery. | Climate and Health Research | **Adaptation** |
| Morrocco | Mtioui et al, 2020 | To assess the direct and indirect carbon emissions of haemodialysis. | Emission assessment | Consumption of electrical energy was the highest (28%) emitter, followed by purchase of equipment and services (27%) and travel of staff and patients (22%). | Climate and Health Risk and GHG Emissions Assessment | **Mitigation** |
|  | Nourdine and Saad, 2021 | To investigate the efficiency, cost and environmental impact of using hybrid renewable energy systems in healthcare. | Modelling | Electricity mix generation reduced carbon emissions. Connection to the grid for excess electricity injection contributed most to lowering emissions. | Climate Smart Health Workforce | **Mitigation** |
| Mozambique | Rossi et al, 2024 | To analyse the impact climate-induced events on healthcare services case study. | Perspective | ~40% of mobile health unit outreach programs halted due to damaged roads. Long walking distances change healthcare seeking behaviour and traditional health practitioners became the first point of care in communities. | Climate-Resilient and Sustainable Technologies and Infrastructure and Supply Chain | **Adaptation** |
|  | Muleia et al, 2024 | To assess health sector vulnerability and adaption needs. | Adaptation assessment | Most regions in the country lacked adaptive capacity and hade an insufficient work force to support health service demand. | Climate and Health Risk and GHG Emissions Assessment | **Adaptation** |
| Nigeria | Olatomiwa, 2016 | To determine the suitability of multiple renewable sources for rural healthcare facilities. | Modelling | Hybrid renewable systems offered lower costs and emissions compared to diesel-only energy generation. | Climate-Resilient and Sustainable Technologies and Infrastructure and Supply Chain | **Mitigation** |
|  | Omodero, 2022 | To assess the extent of climate change-related healthcare costs. | Modelling | There is a positive relationship between carbon emissions and health expenditure to address climate-related ailments. | Climate-Resilient and Sustainable Technologies and Infrastructure and Supply Chain | **Mitigation** |
|  | Oni, 2021 | To evaluate an intervention design to improve health, address climate risks, and promote resilience. | Perspective | Community-based co-produced solutions are required to reduce the impact of floods on healthcare infrastructure, create early warning systems and educate healthcare staff to be disaster-responsive. | Climate-Resilient and Sustainable Technologies and Infrastructure and Supply Chain | **Adaptation** |
|  | Shiyanbade, 2023 | To examine the effectiveness of local governance to mitigate climate change impacts on healthcare. | Mixed methods | Ineffective local governance to mitigate the impacts of climate change is due to inadequate funding, lack of constitutional power, poor leadership. | Climate-Transformative Leadership and Governance | **Adaptation** |
|  | Achirgbenda, Kuhe & Okoli, 2024 | To determine the techno-economic feasibility of a hybrid energy system. | Modelling | Using renewable energy technologies coupled with diesel generator resulted in reduction of harmful gases. | Climate-Resilient and Sustainable Technologies and Infrastructure and Supply Chain | **Mitigation** |
|  | Babatunde et al, 2018 | To provide a life cycle economic and environmental analysis. | Modelling | The solar PV energy system can avoid the ~9000 kg of carbon dioxide emissions. | Climate-Resilient and Sustainable Technologies and Infrastructure and Supply Chain | **Mitigation** |
|  | Ighravwe et al, 2018 | To determine the energy and environmental efficiency of hybrid renewable energy systems. | Modelling | GHG emissions reduced by 64-74% by using hybrid photovoltaic energy systems compared to diesel. | Climate-Resilient and Sustainable Technologies and Infrastructure and Supply Chain | **Mitigation** |
| Rwanda | Nsengiyumva et al, 2020 | To assess nurses' and midwives' climate change awareness with neonatal health. | Cross sectional | Majority (60.4%) of participants had low awareness about climate change, with school being the major contributor to climate knowledge. | Climate Smart Health Workforce | **Adaptation** |
| Senegal | Diallo and Ridde, 2023 | To assess the resilience of a health system against high climate variability. | Interviews | Floods block access to care but also increases care burden for inpatients who cannot be discharged. from road infrastructure blockage. Traditional health workers are mobilised to assist with community care. | Emergency Preparedness and Management | **Adaptation** |
| Somalia | Dursun, Aykut and Dursun, 2021 | To assess the energy efficiency and environmental impact of renewable energy in a hospital. | Modelling | PV/wind/diesel/battery system produced the lowest CO₂ emissions. | Climate resilient and low carbon infrastructure, technologies, and supply chain | **Mitigation** |
| South Africa | Kim and Roodt, 2023 | To reduce theatre’s carbon emissions by implementing a low-flow anaesthesia protocol. | Emission assessment | Implementation of a low-flow anaesthesia protocol reduced volatile gas use by 25% and carbon emissions by 29%. | Integrated Risk Monitoring and Early Warning, and GHG Emission Tracking | **Mitigation** |
|  | Alao, Popoola and Ayodele, 2021 | To determine the ideal biogas-fuelled prime mover for combined heat and power. | Modelling | Fuel cells were the most ideal primer (co-benefits of ease of operation, low emissions, and production of high-grade thermal energy) although with high costs. | Climate-Resilient and Sustainable Technologies and Infrastructure and Supply Chain | **Mitigation** |
|  | dos Santos et al, 2019 | To explore workforce capacity to manage climate change. | Interviews | Immediate and urgent healthcare needs limit the capacity of the facilities to plan for climate-associated consequences. | Climate-Resilient and Sustainable Technologies and Infrastructure and Supply Chain | **Adaptation** |
|  | Frewen, Grossman and Basson, 2022 | To determine the attitudes and knowledge of South African anaesthetists toward environmentally sustainable anaesthetic practice. | Cross sectional | 27% of anaesthesiologists considered the environmental impact when choosing volatile anaesthetic agents, while 24.0% were entirely unaware of the environmental impact of these agents. | Climate Smart Health Workforce | **Adaptation** |
|  | Giuricich, Kleyenstuber and Jeggo, 2024 | Explore the impact of anaesthesia on climate change. | Narrative review | South Africa lacks formal guidelines on sustainable anaesthesia. SASA recommends limiting N₂O and desflurane use and prioritising low-impact techniques. | Climate-Resilient and Sustainable Technologies and Infrastructure and Supply Chain | **Adaptation** |
|  | Irlam et al, 2023 | To determine the knowledge, perceptions of climate change and environmental sustainability of ungraduated health sciences students. | Cross-sectional | Majority were aware of climate change but with limited understanding. Internet and social media are highest source of knowledge. | Climate Smart Health Workforce | **Adaptation** |
|  | Irlam et al, 2023 | To determine the integration of PH and ES in South African health sciences faculties. | Cross-sectional | 53.7% of respondents stated that PH and ES do not form part of any course content. Climate-related activities and courses were more available at post-graduate level. | Climate Smart Health Workforce | **Adaptation** |
|  | Manyuchi, Vogel and Wright, 2023 | To assess climate and heat-health services in a South African district. | Interviews | Services such as climate health information are limited or non-existent. | Climate-Informed Health Programs | **Adaptation** |
|  | Patel, 2024 | To discuss the current landscape of sustainable practices in radiology. | Editorial | Greenhouse gases include manufacture and transport, high-energy consuming equipment, cooling systems, computer workstations, data generation and data storage. | Climate-Resilient and Sustainable Technologies and Infrastructure and Supply Chain | **Mitigation** |
|  | Vorster, 2024 | To determine sustainability practices of South African anaesthesia providers. | Review | Anaesthesiologists use volatile gases without N2O as a first-choice intervention. Experience, level of expertise and working in public/private sector influences choice. | Climate-Resilient and Sustainable Technologies and Infrastructure and Supply Chain | **Mitigation** |
|  | Lister et al, 2022 | To investigate healthcare professionals’ knowledge, attitudes, practices, and barriers to environmental sustainability. | Mixed methods | 60% consider knowledge on sustainability to be limited and don’t consider it in daily practice. 41% implementing sustainability practices in their clinical setting. Barriers include knowledge, finance, lack of buy-in. | Climate Smart Health Workforce | **Adaptation** |
|  | Naidoo, Manyangadze and Lokotola, 2022 | To report on the impact of floods on primary health care facilities. | Report | Floods disrupted travel to healthcare facilities for patients and staff - mobile healthcare units were deployed. Disaster plans don’t make provision or measures for floods. | Emergency Preparedness and Management | **Adaptation** |
|  | dos Santos, 2022 | To explore expert perspectives on climate change and health. | Interviews | High internal temperatures and long waiting times deter patients from seeking care. | Climate and Health Risk and GHG Emissions Assessment | **Adaptation** |
|  | Chersich, 2019 | To determine the extent of South Africa’s health sectors climate change adaptation response. | Review | Climate policies lack health-specific focus and fragile health systems are vulnerable to climate shock events; impacting staff and patient wellness. | Emergency Preparedness and Management | **Adaptation** |
|  | Wright et al, 2019 | To provide an overview of climate, heat and health research priorities. | Report | Research trends have focused on the impact of heat on indoor temperature and morbidity and mortality; the use of early warning systems to predict and manage climate-diseases. | Emergency Preparedness and Management | **Adaptation** |
|  | Weiman, 2017 | To highlight sustainability efforts of a tertiary hospital. | Energy-use assessment | The hospital reduced its coal consumption by 48%. Management played stewardship role to encourage healthcare staff to become climate change agents. | Climate-Informed Health Research | **Mitigation** |
| sub-Saharan Africa | Kilungo 2024 | To provide an overview of climate change and health research in SSA. | Scoping Review (pre-print) | Most climate and health are studies conducted without funding and few receive local funding. | Health and Climate Research | **Adaptation** |
| Uganda | Bishop-Williams et al, 2019 | Investigate associations of seasonal and meteorological parameters with hospital admission counts. | Modelling | High temperatures days were associated increase heat-related hospital service demands. | Health and Climate Research | **Adaptation** |
|  | Felice et al, 2023§ | Investigate the economic feasibility of reducing and replacing diesel generators with greener energy in Lacor Hospital’s energy community. | Modelling | The modelled energy consumption systems reduced carbon emissions between 5% - 74%. | Climate-Resilient and Sustainable Technologies and Infrastructure and Supply Chain | **Mitigation** |
| Zambia | Mroz, 2023 | To determine the impacts of flooding for pregnant women. | Modelling | Flooding severely reduces women's access to maternity care and negatively impacts referral systems during flooding. | Climate and Health Risk and GHG Emissions Assessment | **Adaptation** |
| Zimbabwe | Ray, Goronga, Chigiya and Madzimbamuto, 2022 | To explore the potential role of primary health care (PHC) in disaster risk management. | Report | PHC’s should collaborate with community members to integrate Indigenous Knowledge Systems into education and early warning systems. | Health and Climate Research | **Adaptation** |
|  | Sithole et al, 2021 | To determine the preparedness of the facilities to respond to drought effects. | Report | The healthcare workforce is not adequately trained to manage acute malnutrition; screening and reporting by village health workers was inconsistent. | Climate Smart Health Workforce | **Adaptation** |
|  | Ngwenya B, Oosthuizen J, Cross M, 2018 | To explore the knowledge and awareness of health providers and policy makers regarding climate change health effects | Interviews | There is an absence of heat stress policies, disaster plans, and monitoring; government warning systems prioritised floods over heat. Health officers lacked capacity and training. | Health and Climate Research | **Adaptation** |
| Multinational: Somalia, Kenya, Ethiopia | Lindvall, 2020 | To investigate the health impacts of drought-related migration and health care access in Somalia, Kenya and Ethiopia | Interviews | Healthcare services lack mental health and gender-based violent care. There is lack of disaggregated services for migrants and locals to avoid inequality and potential conflict. | Climate and Health Risk and GHG Emissions Assessment | **Adaptation** |
| Multinational: Botswana, Mauritania, Niger, Nigeria and Togo | Anderson, 2023 | To report on an emergency preparedness and response initiative in sub-Saharan Africa. | Report | A rapid-response teams of specialists were trained to respond to extreme weather event emergencies within 24 hours. | Emergency Preparedness and Management | **Adaptation** |
| Multinational: Benin, Botswana, Cameroon, Cote d'Ivoire, Democratic Republic of Congo, Egypt, Ethiopia, Ghana, Kenya, Namibia, Nigeria, Rwanda, Somalia, South Africa, Sudan, Uganda, Zambia | Zolo et al, 2023 | To assess the state of sustainability education in current medical students and surgical trainees' curricula. | Cross-sectional | 60.3% of students report not getting sustainability education in surgical training. | Climate Smart Health Workforce | **Adaptation** |
| Multinational: Ethiopia, Uganda, South Africa, Ghana, Cameroon, Nigeria | Samba et al, 2023 | To investigate the impact of climate change on nurses and patients in oncology. | Interviews | Climate shock events disrupted patient transport and facility power and caused abandonment of care. | Climate Smart Health Workforce | **Adaptation** |
| Multinational: Nigeria, Democratic Republic of Congo, Uganda, Kenya, Multinational: Zimbabwe, South Africa, Sudan, Rwanda, Zambia, Botswana, Malawi, Somaliland | Scheerens et al, 2021 | To explore family physicians perceived perspectives on climate change, health and migration | Focus group discussions | Young clinicians are unable to accurately diagnosis climate-related diseases. Climate-related migration has impact on resources and capacity of hosting/receiving country. | Climate Smart Health Workforce | **Adaptation** |
| Multinational: Kenya, Tanzania | Baddley and Rasheed, 2023 | To demonstrate an approach for measuring hospital carbon footprint in supply chain. | Modelling | Scope 3 emissions dominated, from pharmaceuticals, surgical supplies, and lab consumables. Supply chain emissions were high (78–96%). | Climate and Health Risk and GHG Emissions Assessment | **Mitigation** |
| Multinational: Mozambique, Ethiopia | Quinn, 2022 | To explore how the potential of multi-sectoral climate-health response. | Perspective | A multi-sectoral observatory was established to link meteorological and health data for decision-making in Mozambique, while in Ethiopia, a malaria early warning system was developed at district level. | Integrated risks monitoring, early warning, and emissions tracking | **Adaptation** |
| Multinational: Egypt, Nigeria | Rawashdeh et al, 2024 | Determine radiographers’ perceptions, practices and barriers to environmental sustainability. | Cross sectional | Common sustainable practices included low-energy lighting, power monitoring, efficient heating, cycling, public transport use and emission-reduction policies. Reported barriers were lack of leadership, staff attitudes, and insufficient training. | Climate Smart Health Workforce | **Adaptation** |
| Africa | Negev et al, 2018 | To provide recommendations for how healthcare systems can adapt to climate-related migration. | Review | Train health professionals; involve migrants in healthcare system governance to ensure culturally appropriate and bias-free care. | Health and Climate Research | **Adaptation** |
|  | Aron, 2006 | To present barriers of using geo-spatial data for public health adaptation to climate change. | Review | Not all geographic areas on SSA are well suited for accurate and reliable early warning systems. | Integrated Risk Monitoring and Early Warning, and GHG Emission Tracking | **Adaptation** |
|  | Striebig, 2012 | To develop an interactively design tool for sustainable health clinic infrastructure | Modelling | Use of solar energy, rainwater harvesting, green roofing, and passive ventilation significantly reduces the clinic’s environmental footprint. | Climate-Resilient and Sustainable Technologies and Infrastructure and Supply Chain | **Mitigation** |
|  | Wright et al, 2024 | To describe climatic changes facing Africa and identify strategies to reduce adverse effects. | Review | Adaptation activities include early warning alert and response systems for health surveillance. Renewable energy options could support access to resources and services in times of climate shocks. | Health and Climate Research | **Adaptation** |
|  | Atela et al, 2024 | To understand how collaborations can facilitate the integration of health considerations into climate change policies and systems. | Mixed methods | Of 188 mapped African stakeholders, only 97 worked at the climate–health nexus; many face funding, vulnerability, and geopolitical constraints. | Health and Climate Research | **Adaptation** |
|  | Alzaabi et al, 2023 | To quantify the carbon footprint of SABA and controller inhalers. | Retrospective cohort study | Short-acting β2-agonist (SABA) inhalers account for ~70% of inhaler-related greenhouse gas (GHG) emissions. | Integrated Risk Monitoring and Early Warning, and GHG Emission Tracking | **Mitigation** |
|  | Irlam, Scheerens and Mash, 2023 | To describe the integration of planetary health (PH), into health professions curricula in Africa. | Position paper | Need to create platforms for knowledge, collaboration and best practices for exchange among educator, including how faculties can measure their environmental footprints. | Climate Smart Health Workforce | **Adaptation** |
|  | Chersich et al, 2023 | To describe approaches to address heat-related maternal and child health. | Narrative review | Interventions should be designed in collaboration with pregnant and post-partum women, health workers, male partners. Need for heat health pre- and in-service training for healthcare workers. | Climate-informed Health Research | **Adaptation** |
|  | Rublee et al, 2020 | To review the outcomes of an emergency medicine climate change education and advocacy conference. | Commentary | Priorities include integration of climate change into academic curriculum, developmental research agenda on health climate change nexus, and inclusion of healthcare professionals in health climate change policy formulation. | Climate Smart Health Workforce | **Adaptation** |
|  | Wright et al, 2021 | To advocate for regional intergovernmental collaboration to address climate change and health adaption. | Communication | Climate change and migration require cross-border health governance on service delivery, access to medicines, health communication and early warning systems for disease outbreaks. | Health and Climate Research | **Adaptation** |
| North Africa | Al-Adawi, 2022 | To address the absence and role of healthcare workers in addressing climate change. | Editorial | Healthcare workers have a responsibility to be the voice of climate change response. This requires incorporating climate change into curriculum and research. | Health and Climate Research | **Adaptation** |
