# Supplementary material for: Climate change adaptation and mitigation in African health systems: a scoping review of research and policy translation
Source: BMJ Open. 2026 Sep 21;16(9):e119188. doi: 10.1136/bmjopen-2026-119188 (PMC13599907; doi:10.1136/bmjopen-2026-119188)
Supplement: Supplementary file 5 [file bmjopen-16-9-s004.docx]

**Supplemental Table 3: Characteristics of the included grey literature publications (WHO Domain: Climate transformative leadership and governance)**

| **Country** | **Title / Year** | **Document type** | **Health sector role** | **Notable features** |
| --- | --- | --- | --- | --- |
| **Multi-sectoral documents that include the health system** | | | | |
| South Africa | Climate Change Act (2024) | Law | Requires health sector to develop adaptation plans and emission targets | Ministerial oversight.  Mitigation: GHG targets, inventories, carbon budgets, phase-out of synthetic gases.  Adaptation: National, provincial & municipal strategies aligned with NAS; reporting & coordination; resilience across social, economic & environmental systems. |
| Mauritius | Climate Change Act (2020) | Law | Health sector obligated to prepare adaptation strategies | Mitigation: GHG cuts in energy/transport/industry, carbon sinks, renewables, green economy.  Adaptation: sectoral resilience, vulnerability assessments, risk reduction, awareness. |
| Botswana | Climate Change Policy (2021) | Policy | Research and evidence-based decision-making | Climate change threatens health directly (heat, drought, floods) and indirectly (water- and vector-borne diseases, malnutrition). Priorities include research on health impacts of extreme weather, strengthening community involvement in climate-related health resilience. |
| Lesotho | Climate Change Policy (2017) | Policy | Data management, surveillance, coordination | Priorities include integration of climate-health risks in policy; education on climate–health nexus; strengthening surveillance & early warning systems; improve data sharing; foster cross-sector partnerships; map disease/vulnerable groups; enhance intersectoral coordination. |
| Namibia | Climate Change Policy (2011) | Policy | WASH in hospitals, access to care during floods | Priorities include access to safe water & sanitation in affected areas/public facilities; provision of medical assistance for climate-related diseases & malnutrition. |
| Malawi | Climate Change Policy (2016) | Policy | Limited health mention | Priorities include mainstreaming HIV/AIDS into climate change planning. |
| Zambia | Climate Change Policy (2016) | Policy | Rehabilitation of health infrastructure | Climate change recognised in health policy goals with focus on community healthcare and health-risk awareness. |
| Angola | National Climate Change Strategy (2021) | Strategy | Disease monitoring, early warning, emergency preparedness | Health adaptation: tropical disease prevention/monitoring and sanitation through multisectoral collaboration. |
| Zimbabwe | National Climate Change Response Strategy (2019) | Strategy | Build resilience against climate-sensitive diseases | Priorities include strengthening health surveillance. Cost estimations for health sector adaptation are included. |
| Tanzania | Climate Change Strategy (2021) | Strategy | Workforce education, integration of climate into training | Multi-sectoral approach required to address climate-related health impacts. |
| Botswana | Climate Change Action Plan (2018) | Strategy | Workforce capacity, measurable indicators | Priorities include training 90% of health workers on climate change by 2030. |
| **Health system-specific documents** | | | | |
| Madagascar | Health National Adaptation Plan (2018) | Plan | Comprehensive adaptation actions; surveillance, emergency preparedness | Seven components for priority: risk & capacity assessment; capacity building; integrated environment health surveillance & early warning systems; research; monitoring & evaluation; governance/coordination. |
| South Africa | Health National Adaptation Plan (2014–2019) | Plan | Governance, risk assessment, research | Priorities included developing indicators, resource mobilisation, high‑risk area identification, alignment with social determinants, intersectoral collaboration; strengthen early warning and emergency preparedness. |
| Tanzania | Health National Adaptation Plan (2018) | Plan | Surveillance, community awareness | Priorities from V&A: vector-borne diseases, water-related diseases, nutrition; floods & droughts. Need to mainstreams climate risks into health policies/HMIS; climate‑sensitive early warning for outbreaks; investments in resilient facilities, technologies and WASH. |
| Zambia | Health National Adaptation Plan (2017) | Plan | Climate-sensitive risk profiling | V&A assessment priorities: focus on malaria, diarrheal, respiratory, recurrent cholera; links to WASH and nutrition, adaptation mainstreaming into public health programmes. |
